# Mblk-1/E93, an ecdysone related-transcription factor, targets synaptic plasticity-related genes in the honey bee mushroom bodies

**DOI:** 10.1038/s41598-022-23329-z

**Published:** 2022-12-09

**Authors:** Yasuhiro Matsumura, Taiko Kim To, Takekazu Kunieda, Hiroki Kohno, Tetsuji Kakutani, Takeo Kubo

**Affiliations:** Department of Biological Sciences, Graduate School of Science, The University of Tokyo, Bunkyo-ku, Tokyo, Japan

**Keywords:** Evolution, Molecular biology, Neuroscience

## Abstract

Among hymenopteran insects, aculeate species such as bees, ants, and wasps have enlarged and morphologically elaborate mushroom bodies (MBs), a higher-order brain center in the insect, implying their relationship with the advanced behavioral traits of aculeate species. The molecular bases leading to the acquisition of complicated MB functions, however, remains unclear. We previously reported the constitutive and MB-preferential expression of an ecdysone-signaling related transcription factor, Mblk-1/E93, in the honey bee brain. Here, we searched for target genes of Mblk-1 in the worker honey bee MBs using chromatin immunoprecipitation sequence analyses and found that Mblk-1 targets several genes involved in synaptic plasticity, learning, and memory abilities. We also demonstrated that Mblk-1 expression is self-regulated via Mblk-1-binding sites, which are located upstream of *Mblk-1*. Furthermore, we showed that the number of the Mblk-1-binding motif located upstream of *Mblk-1* homologs increased associated with evolution of hymenopteran insects. Our findings suggest that *Mblk-1*, which has been focused on as a developmental gene transiently induced by ecdysone, has acquired a novel expression pattern to play a role in synaptic plasticity in honey bee MBs, raising a possibility that molecular evolution of Mblk-1 may have partly contributed to the elaboration of MB function in insects.

## Introduction

In insects, mushroom bodies (MBs), a higher-order center in insect brains, play important roles in olfactory associative learning^1^, spatial learning^2^, sensory integration^3^, and social behavior^4^. In Hymenoptera, aculeata species such as bees, ants, and wasps have enlarged and morphologically elaborated MBs compared to the most primitive hymenopteran insects like sawflies, suggesting a relationship between MB elaboration and the advanced behavioral traits of these insects^5,6^. The molecular and neural bases underlying how aculeate MBs acquired elaborated functions, however, remain elusive. Aculeate hymenopteran species show advanced social behaviors^7,8^. For example, European honey bee (*Apis mellifera* L.) workers perform the ‘waggle dance’ to inform their nestmates of the location of food sources that they memorize during foraging flights^9^. Therefore, honey bees are useful model insects for understanding neural mechanisms underlying sociality, learning ability^10,11^ and brain evolution in insects.

The honey bee MBs comprise 4 types of interneurons termed Kenyon Cells (KCs): class I large-, middle-, small-type KCs, and class II KCs, which are distinguished on the basis of their location, the size of their somata, and their gene expression profiles^12^. Among these KC subtypes, large-type KCs (lKCs) are characteristic in that they preferentially express genes involved in Ca^2+^-signaling, including *Ca*^*2*+^*/calmodulin-dependent protein kinase II* (*CaMKII*)^13,14^. Since Ca^2+^-signaling pathways play an important role in synaptic plasticity, the primary mechanism of learning and memory functions in many animal species^15^, it is plausible that lKCs are involved in the learning and memory abilities that underlie honey bee social behaviors.

In addition to the genes involved in Ca^2+^-signaling, a sequence-specific transcription factor, termed *mushroom body large-type Kenyon-cell specific protein-1* (*Mblk-1*)/*ecdysone-induced protein 93F* (*E93*) (hereafter termed as *Mblk-1*) is preferentially expressed in lKCs^16–18^. *Mblk-1* is widely conserved in animal species from invertebrates to vertebrates^19^. Especially in insects, *E93*, the *Mblk-1* homolog, is upregulated by the molting hormone ecdysone and ecdysone receptor and triggers programmed cell death of larval tissues during metamorphosis^20–24^. In *Drosophila melanogaster*, *E93* (*DmE93*) is expressed in MB neuroblasts during later pupal stages where it activates autophagy to eliminate the neuroblasts^24^. In the honey bee, the preferential expression of *Mblk-1* in lKCs suggests its important role in the function of lKCs, but the function of Mblk-1 as a transcription factor and its target genes in honey bee lKCs are unknown.

We previously used an affinity-purified antibody raised against a partial recombinant Mblk-1 protein to examine Mblk-1 protein expression in the honey bee brain^18^. In the present study, we performed chromatin immunoprecipitation sequencing (ChIP-seq) using this antibody to identify Mblk-1 target genes in honey bee brains. We found that Mblk-1 regulates genes related to synaptic plasticity, such as *CaMKII*^25–27^ and *Mblk-1* itself, suggesting that Mblk-1 has acquired a new expression mechanism and roles related to synaptic plasticity in the honey bee MBs. We propose that acquisition of unconventional functions of Mblk-1 in lKCs might have partly contributed to the evolution of learning and memory abilities and social behaviors in aculeate Hymenoptera.

## Methods

### Insects

European honey bee colonies maintained at The University of Tokyo (Hongo Campus) were used. Some colonies were also purchased from a local dealer (Kumagaya Honeybee Farm, Saitama, Japan). For ChIP-seq analysis using adult worker samples, we randomly collected workers from the hives irrespective of age and/or role. For ChIP-seq analysis using pupal worker samples, we selected and used worker pupae with brown or purple eyes (stages: P3-P5). Sawflies (*Athalia rosae*) were a gift from Mr. Takayoshi Kuwabara. Adult sawflies within 10 days after emergence were used for the experiment.

### Analysis of genes expressed in adult worker MBs using RNA-seq data

The RNA-seq data (low-value reward and no-dance) was obtained from GEO accession: GSM3747967^28^. The value of 2012_309_TTAGGC_L007_Aligned.sortedByCoord.out.bam in GSM3747967_2012_309_TTAGGC_L007_Aligned.sortedByCoord.out.bam.counts.txt was divided by each gene length, and the top 40 genes with the highest divided values were displayed.

### Chromatin preparation

80 MBs dissected from adult workers or 60 whole brains dissected from worker pupae were used per single chromatin immunoprecipitation sequence (ChIP-seq) experiment. The ChIP-seq experiments were conducted using 2 biological replicates for the adult and pupal samples. The MBs or whole brains were dissected under binocular microscopy from cooled and anesthetized honey bees and homogenized with plastic pestles in 200 µl of buffered insect saline (20 mM Tris–HCl [pH 7.4], 130 mM NaCl, 5 mM KCl, 1 mM CaCl_2_, 10 mM HEPES buffer, protease inhibitor cocktail [Roche], and PhosSTOP [Roche]). The cells were fixed with formaldehyde (1% final conc.) for 12 min at room temperature. The fixing reaction was quenched with 2.5 M glycine (125 mM final conc.) for 10 min on ice. The cells were washed 3 times with 150 µl of buffered insect saline and centrifuged (600×*g*, 5 min, 4 °C). The supernatant was removed, and the cells were suspended in 300 µl of buffered insect saline. The cells were crushed for 25 s using zirconium beads (TOMY) and Micro Smash MS-100 (TOMY). After centrifugation, the supernatant was discarded and 200 µl of nuclei lysis solution (10% glycerol, 0.5% NP-40, 2 mM EDTA, 50 mM Tris–HCl [pH 7.4], and protease inhibitor cocktail [Roche]) were added. The sample was incubated on ice for 30 min. After centrifugation, the supernatant was discarded and 500 µl of Micrococcal Nuclease buffer (50 mM Tris–HCl [pH 7.4] and 5 mM CaCl_2_) was added. The sample was kept on ice for 10 min and centrifuged. After the supernatant was discarded, the pellet was resuspended in 140 µl of Micrococcal Nuclease buffer containing protease inhibitor cocktail and 0.2 µl of Micrococcal Nuclease (2000 gel units/µl, New England BioLabs). The sample was incubated at 37 °C for 12 min and digestion was stopped by 500 mM EDTA (total volume: 150 µl). After adding 300 µl of 1.5 × ChIP dilution buffer (1 × ChIP dilution buffer: 50 mM Tris–HCl [pH 7.4], 167 mM NaCl, 1.1% Triton X-100, 0.11% SDS), the sample was sonicated (output 10%, 12 s at 60 s intervals, 3 times) using a SONIFIER250 (Branson). After sonication, the sample was centrifuged, and the supernatant was collected for immunoprecipitation.

### Chromatin immunoprecipitation (ChIP)

The sample containing the fragmented chromatin and anti-Mblk-1 antibody-attached Dynabeads Protein A (Invitrogen) were incubated with rotation for 45 min at 4 °C. The Dynabeads complex was washed twice with RIPA buffer (50 mM Tris–HCl [pH 7.4], 150 mM NaCl, 1 mM EDTA, 0.1% SDS, 1% Triton X-100), and 3 times with Phosphate-Buffered Saline (PBS). The washed Dynabeads complex was resuspended in PBS. The solution containing the beads was transferred to a new clean tube. The tube was placed on magnet and the supernatant was removed. The anti-Mblk-1 antibody and protein complex was eluted by elution buffer (1% SDS, 50 mM Tris–HCl [pH 7.4], 8.5 mM EDTA). The tube was placed on magnet and the supernatant was transferred to a new tube. After adding TE buffer and 10 mg/ml RNase A, the sample was incubated for 1 h at 50 °C, and then 20 mg/ml proteinase K was added, and the crosslink was reversed by incubation for 12 h at 50 °C. The DNA was purified and processed to sequencing preparation. The sequencing library was prepared according to the Illumina protocol and sequenced on an Illumina Hiseq2500.

### ChIP-seq data processing

Sequence reads were aligned to the reference genome (Amel_HAv3.1)^29^ using Bowtie2 with default parameters^30^. Peak calling was performed using MACS2^31^ and the input DNA sample as a control. After peak calling, we visualized the ChIP-seq data using the Integrative Genomics Viewer (IGV)^32^. The ChIP-seq experiments were performed twice using 2 biological replicates. The peak signals detected in the 2 replicates were considered reproducible if they were located within 1 kb in the genome. Subsequent analysis was conducted using the signals that were reproducible.

### GO enrichment analysis

Taking advantage of the well-characterized GO annotations for *Drosophila melanogaster* genes, we first assigned a *Drosophila melanogaster* ortholog for each *Apis mellifera* protein by reciprocal BLAST searches between all *Apis mellifera* protein sequences (Amel_HAv3.1) and all *Drosophila melanogaster* protein sequences (UP000000803 in UniProt). The *Drosophila melanogaster* protein with the reciprocal best hit was considered the ortholog for each *Apis mellifera* protein. Genes unique to *Apis mellifera* and/or genes with no corresponding *Drosophila melanogaster* homologs were not used for further analysis. We performed GO enrichment analysis using the assigned *Drosophila melanogaster* homologs and Metascape^33^.

### Motif analysis

Sequences of ± 150 bp (300 bp) from the centers of the reproducible signals with a peak calling q-value threshold of 0.001 in the analysis of adult MBs were obtained. These sequences were inputted into MEME-ChIP (Version 5.2.0), which discovers novel DNA-binding motifs with MEME and DREME^34^. As the motif width options, -minw was set to 6 and -maxw was set to 17. No control sequences were used for MEME. The positive sequences were shuffled to create the control set for DREME.

### Luciferase assay

We obtained the coding sequence of *Mblk-1* from Mblk-1/pPac-PL^35^ and inserted it into a multiple cloning region of an expression vector, pHEK293 Ultra Expression Vector II (TaKaRa). Reporter vectors were constructed by inserting 6xGAGA, 6xGAGA with a 4-bp spacer sequence, 6xATTTTG, 6xGAAATTTT, 6xATTTTGG, and an Mblk-1-binding region sequence (NC_037652.1:7063408-7063595, 188 bp), respectively, into the upstream region of minimal promoter of pGL4.23 [luc2/minp]. For the Mblk-1-binding region sequence, we used the overlap region of the 2 Mblk-1-binding sequences (NC_037652.1:7063147-7063709 and NC_037652.1:7063400-7063629) obtained from the 2 independent ChIP-seq experiments. pRL-TK was used as an internal control vector to normalize transfection efficiency. The Mblk-1 expression vector or pHEK 293 Ultra Expression Vector II without the Mblk-1 coding sequence as a negative control, pHEK293 Enhancer Vector, one of the reporter vectors (pGL4.23 with the motif sequences), and the internal control vector (pRL-TK) were transfected to HEK293T cells (RCB2202) from RIKEN BioResource Center using Lipofectamine LTX Reagent with PLUS Reagent (Invitrogen). The pHEK293 Enhancer Vector was used to enhance the expression of *Mblk-1* in pHEK293 Ultra Expression Vector II. We measured *Photinus pyralis* (firefly) and *Renilla reniformis* luciferase activities 40–48 h after transfection with a Dual-Luciferase Reporter Assay System (Promega) and 2030 ARVO X5 (PerkinElmer). *Photinus pyralis* (firefly) luciferase activity was first normalized with the co-transfected internal control luciferase activity and then values of normalized luciferase activity of the control assay using the pHEK 293 Ultra Expression Vector II without the Mblk-1 coding sequence were subtracted to exclude effects other than those of Mblk-1 expression. The obtained value is shown as relative luminescence on the vertical axis of the graphs. We compared the values from vectors containing candidate sequences with that of the control vector (pGL4.23) to determine how the sequences affect the expression of *luciferase*.

### Single-cell RNA-seq data analysis

We obtained single-cell RNA-seq data from the previously reported GEO accession ID GSE130785^36^. Using Seurat Version 4.1.1^37^, we selected cells for clustering under the following conditions (min.cells = 3 and min.features = 200 and nCount_RNA > 1000 and nFeature_RNA > 500). After SCTransform normalization, we performed cell clustering (PC = 30 and resolution = 0.5). Doublet cells were detected using DoubletFinder (doubletFinder_v3(PCs = 1:30, pN = 0.25, pK = 0.01, nExp = nExp_poi.adj, reuse.pANN = FALSE, sct = TRUE))^38^. Cells determined to be doublets were removed and marker genes for each cluster were found by FindAllMarkers. Marker genes with an adjusted p-value under 0.05 were used for further analyses.

### RNA probe synthesis and in situ hybridization

Total RNA was extracted from 3 honey bee adult worker brains using TRIzol Reagent (Invitrogen). After removing the genomic DNA with the gDNA Eraser from the PrimeScript RT reagent Kit with gDNA Eraser (Perfect Real Time), the extracted RNA was reverse-transcribed into cDNA using the same kit. The cDNA was partially amplified by PCR using Ex *Taq* (TaKaRa) with primers specific for each gene. The PCR products were cloned using pGEM-T Easy Vector Systems (Promega). The primer sequences used to synthesize the in situ hybridization RNA probes are listed in Supplementary Table 1. The DNA templates for in vitro transcription were amplified by PCR with M13 forward and reverse primers. The digoxigenin (DIG)-labeled sense and antisense RNA probes were synthesized using the amplified DNA template, a DIG RNA Labeling Mix (Roche), SP6 RNA polymerase (Roche), and T7 RNA polymerase (Roche). For in situ hybridization, dissected whole brains were embedded in Tissue-Tek OCT compound (Sakura Finetek Japan) without fixation and immediately frozen and stored at − 80 °C until use. The frozen whole brains were sliced into 10-µm thick sections. In situ hybridization was performed essentially as described previously^39^. Images were captured with a light microscope BX-50 (Olympus).

### Quantitative reverse transcription-polymerase chain reaction (qRT-PCR)

Total RNA was extracted from each tissue (brain, head without brain, thorax, and abdomen) using TRIzol Reagent (Invitrogen). cDNA synthesis was performed using a PrimeScript RT reagent Kit with a gDNA Eraser (Perfect Real Time; TaKaRa). qRT-PCR analysis was performed using TB Green Premix Ex *Taq *II (Tli RNaseH plus; Takara) and Light Cycler 480 Instrument II (Roche Life Science, Indianapolis, IN, USA). The following primers were used for gene amplification: *Mblk-1* homolog (LOC105691346), 5′-GCAACAGCAACAGAGAGAAAGG-3′ and 5′-CGTTCCCGTACCGTCATAC-3′; *60S ribosomal protein L32* (LOC105692622), 5′-ACAGAGTTCGTAGGCGCTTC-3′ and 5′-GCATCATCAAGACCTCCAAC-3′. The PCR conditions were as follows: denaturation at 95 °C for 30 s; 45 cycles of PCR (95 °C for 5 s, 61 °C for 15 s, 72 °C for 20 s), melting, 95 °C for 5 s, 65 °C for 60 s, then 95 °C (Ramp Rate: 0.11 °C/s) and cooling, 50 °C for 30 s.

### Analysis of the GAGA motifs upstream of *Mblk-1* homologs

Upstream sequences within 2 kb or 10 kb of the transcription start site of each *Mblk-1* homolog were obtained from NCBI. The numbers of GAGA or GAG sequences in the upstream region of each *Mblk-1* homolog were counted, allowing for overlap. For example, a ‘GAGA’ sequence was counted as one GAGA and one GAG, and a ‘GAGAG’ sequence was counted as one GAGA and two GAGs. The assemblies used were as follows: *Apis mellifera*, Amel_HAv3.1^29^; *Harpegnathos saltator*, Hsal_v8.5^40^; *Solenopsis invicta*, UNIL_Sinv_3.0; *Vespa mandarinia*, V.mandarinia_Nanaimo_p1.0; *Fopius arisanus*, ASM80636v1^41^; *Nasonia vitripennis*, Nvit_psr_1.1^42^; and *Athalia rosae*, Aros_2.0^43^.

### Statistical analysis

Statistical analysis was conducted as indicated in the text and figure legends. Where required P values were adjusted using Bonferroni or Benjamini–Hochberg correction. In Fig. 3D, Tukey–Kramer’s tests after one-way ANOVA were performed (n = 3). F values, P values, and degrees of freedom were 8.09, 0.00831 and 3 (right panel) and 99.3, 2.52 × 10^–5^ and 2 (left panel), respectively. Student’s *t* test used in Fig. 6B was two sided (n = 4). In Fig. 6D, one-way ANOVA was performed. F value was 2.09, P value was 0.180 and degrees of freedom was 3 (n = 3).

## Results

### Identification of Mblk-1 target gene candidates in the worker MBs by ChIP-seq analysis

**Figure 1:**
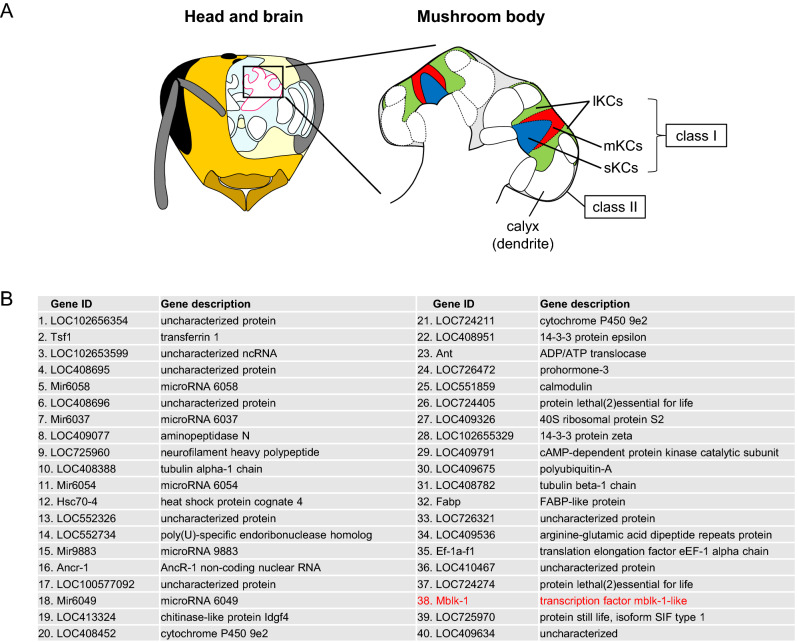
Analysis of genes highly expressed in adult worker MBs. (**A**) Schematic drawings of the head, brain, and MBs of the honey bee. The MBs are a paired structure in the insect brain, and the somata of the 3 class I KC subtypes are localized inside of each calyx, whereas those of class II KCs are localized at the outer surface of the MB calyces. *lKCs,* large-type KCs shown in green; *mKCs,* middle-type KCs shown in red; *sKCs,* small-type KCs shown in blue. (**B**) List of the top 40 genes highly expressed in adult worker MBs. Gene ID and gene description are provided for each gene.

Mblk-1 is expressed in a lKC-preferential manner in adult worker MBs (Fig. 1A)^16,18^. When we searched for highly expressed genes in the honey bee MBs using previously reported RNA-seq data^28^, we found that Mblk-1 was the 38th most highly expressed gene and the most highly expressed among transcription factors in MBs (Fig. 1B), implying its important role in the adult honey bee MBs.

We previously prepared an antibody that binds specifically to Mblk-1 protein (hereafter termed anti-Mblk-1 antibody) (Fig. S1A)^18^. We first tested whether the anti-Mblk-1 antibody could be used for ChIP-seq analysis. In the immunoprecipitated sample, as well as in the input and supernatant samples after immunoprecipitation using worker MB lysate, full-length Mblk-1 protein was mainly detected as a 250-kDa band (Fig. S1B). The molecular weight of Mblk-1 is estimated as 175 kDa based on the protein sequence, but our previous study confirmed that the full-length Mblk-1 protein migrated at the position around 250-kDa in SDS-PAGE analysis^18^. Therefore, the immunoprecipitation results confirmed that the anti-Mblk-1 antibody can be used for immunoprecipitation.

**Figure 2:**
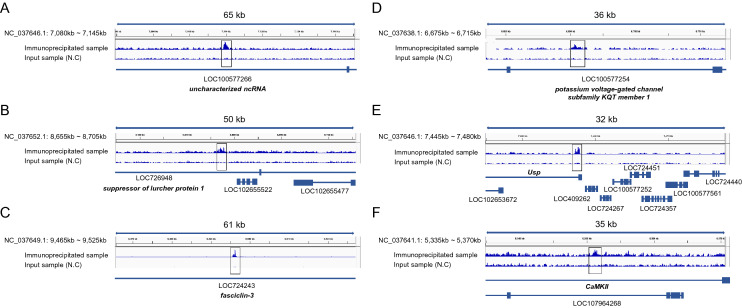
Mblk-1-binding signals and their surrounding genomic regions. The waveforms in each panel show the accumulation of sequence reads in the corresponding genomic region when using samples immunoprecipitated by the anti-Mblk-1 antibody (upper lane), and input samples (lower lane, as negative control). The Mblk-1-binding signals are boxed with black lines in the waveform lanes. Bars and boxes under the waveforms indicate exons and introns, respectively, annotated within the corresponding genomic regions. The gene ID and gene name of each gene are shown below the gene structure. (**A**–**F**) Panels correspond to (**A**) Gene Nos. 1, (**B**) Gene Nos. 2, (**C**) Gene Nos. 4, (**D**) Gene Nos. 9, (**E**) Gene Nos. 14, and (**F**) Gene Nos. 17 listed in Table 1, respectively. Note that there are sometimes overlapping genes for each genomic region, for example, LOC726948, which is annotated as *suppressor of lurcher protein 1*, overlaps with LOC102655522 and LOC102655477.

We performed ChIP-seq analysis using homogenates of adult worker MBs and the anti-Mblk-1 antibody. A total of 593 genes were identified as target gene candidates of Mblk-1, in which Mblk-1-binding signals were located within 10 kb upstream or downstream of the corresponding genes (Table 1, Supplementary Data 1, and 3) when using a peak calling q-value threshold of 0.005 and the input DNA sample as a control. Schematic diagrams of some peak signals and their surrounding Mblk-1 target gene candidates are shown in Fig. 2A–F. The signal with the lowest q-value was in the third intron of an uncharacterized non-coding RNA gene (Gene 1 in Table 1 and Fig. 2A). Genes related to neural function, such as *suppressor of lurcher protein 1* (Gene 2–1 in Table 1 and Fig. 2B), *fasciclin-3* (Gene 4 in Table 1 and Fig. 2C), *potassium voltage-gated channel subfamily KQT member 1* (Gene 9 in Table 1 and Fig. 2D), and *CaMKII* (Gene 17-1 in Table 1 and Fig. 2F) were also identified as Mblk-1 target gene candidates. Moreover, among the Mblk-1 target gene candidates, some ecdysone-signaling pathway-related genes such as *ecdysone-induced protein 75* (*E75*) and *Ultraspiracle* (*Usp*) were identified. This is consistent with the fact that *E75* is a direct regulatory target of *Dm*E93^21^. *Usp* was listed in Table 1, and the peak signal was detected in the 5′ untranslated region of this gene (Fig. 2E).

**Table 1:** Top 20 ChIP-seq peak signals from adult worker MB homogenates and Mblk-1 target gene candidates located within ± 10 kb around the corresponding signals. Gene ID, gene description, signal fold enrichment, − log_10_ (q-value), and signal position relative to the genes are shown for each ChIP-seq peak signal. The dashes for the 5th peak signal indicate the absence of genes located within ± 10 kb of the peak signal. Note that side numbers (e.g., 2-1 and 2-2) indicate that multiple genes are located within ± 10 kb of the corresponding peak (e.g., peak 2).

Gene no.	Gene ID	Gene description	Signal fold enrichment	− log_10_ (q-value)	Signal position	Gene no.	Gene ID	Gene description	Signal fold enrichment	− log_10_ (q-value)	Signal position
1	LOC100577266	Uncharacterized ncRNA	6.45	35.7	Intron	13-1	LOC410920	*Putative inorganic phosphate cotransporter*	5.79	19.4	Intron
2-1	LOC726948	*Suppressor of lurcher protein 1*	7.01	32.4	Intron	13-2	LOC102655189	*Sperm flagellar protein 1-like*			Upstream
2-2	LOC102655522	Uncharacterized ncRNA			Upstream	13-3	LOC113218628	Uncharacterized ncRNA			Downstream
3	LOC552397	*Krueppel-like factor 6*	4.78	32.3	Intron	13-4	LOC725401	*U6 snRNA-associated Sm-like protein LSm3*			Downstream
4	LOC724243	*Fasciclin-3*	3.67	32.1	Intron	13-5	LOC410922	*Wiskott–Aldrich syndrome protein family member 3*			Upstream
5	–	–	6.35	30.1		13-6	LOC102655259	*5-Methylcytosine rRNA methyltransferase NSUN4*			Downstream
6	LOC724450	Uncharacterized	6.42	26.4	Intron	14-1	*USP*	*Ultraspiracle*	4.73	18.1	Intron
7-1	LOC551725	*Importin-7*	6.72	25.8	Intron	14-2	LOC409262	Uncharacterized protein C7orf26 homolog			Upstream
7-2	LOC726708	*F-box only protein 28*			Upstream	14-3	LOC724267	*Ribosomal RNA-processing protein 7 homolog A*			Downstream
7-3	LOC100576242	Cilia- and flagella-associated protein 91			Downstream	14-4	LOC100577252	*Transmembrane protein 145*			Upstream
7-4	LOC726647	Uncharacterized			Downstream	14-5	LOC724451	*FAD-linked sulfhydryl oxidase ALR*			Downstream
7-5	LOC409225	*NEDD8-conjugating enzyme Ubc12*			Downstream	14-6	LOC724357	Uncharacterized			Upstream
7-6	LOC409226	*FAS-associated factor 1*			Upstream	15-1	LOC413051	Uncharacterized protein F13E9.13, mitochondrial	5.31	18.0	Intron
8	LOC724450	Uncharacterized	5.97	25.5	Intron	15-2	LOC725263	*Tumor protein p53-inducible nuclear protein 2*			Upstream
9	LOC100577254	*Potassium voltage-gated channel subfamily KQT member 1*	5.64	23.3	Intron	15-3	LOC100577575	*28S ribosomal protein S15, mitochondrial*			Upstream
10-1	LOC724653	*Intraflagellar transport protein 43 homolog*	6.13	23.2	Downstream	15-4	LOC107964828	*Cuticle protein 19*			Upstream
10-2	LOC102655740	*Mitochondrial import receptor subunit TOM40 homolog 1-like*			Upstream	15-5	LOC725186	*Chromatin modification-related protein MEAF6*			Downstream
10-3	LOC724698	*Isthmin-1*			Downstream	16	LOC100577656	Uncharacterized	4.93	18.0	Intron
10-4	LOC411006	*Endoplasmic reticulum lectin 1*			Downstream	17-1	*Camkii*	*Calcium/calmodulin-dependent protein kinase II*	5.24	17.7	Intron
10-5	LOC100578879	*CASC3*			Upstream	17-2	LOC107964268	Uncharacterized			Intron
10-6	LOC107964328	*Bone morphogenetic protein 10*			Downstream	18	LOC408278	*Tyrosine-protein kinase Drl*	3.30	17.7	Intron
10-7	LOC408789	Uncharacterized			Downstream	19-1	LOC113219117	Uncharacterized ncRNA	5.08	17.5	Intron
11-1	*Err*	*Estrogen-related receptor*	6.63	22.6	Intron	19-2	LOC107965194	Uncharacterized ncRNA			Downstream
11-2	LOC724926	*DNA polymerase alpha subunit B*			Downstream	20	LOC408562	Uncharacterized	4.80	17.4	Intron
11-3		MicroRNA mir-996			Downstream						
11-4		MicroRNA mir-279a			Downstream						
12-1	LOC725575	*PELPK1*	5.25	20.8	Intron						
12-2	LOC102654724	Uncharacterized ncRNA			Upstream						
12-3	LOC100577136	*Diphthine methyltransferase*			Upstream						
12-4	LOC725607	*Cyclin-dependent kinase 2-associated protein 1*			Upstream						
12-5	LOC413069	*H*(+)/*Cl*(*−*)* exchange transporter 7*			Downstream						
12-6	LOC409752	Uncharacterized			Upstream						
12-7	LOC413068	*Intraflagellar transport protein 46 homolog*			Downstream						
12-8	LOC107966062	Uncharacterized			Downstream

### Three distinct DNA binding motifs of Mblk-1

**Figure 3:**
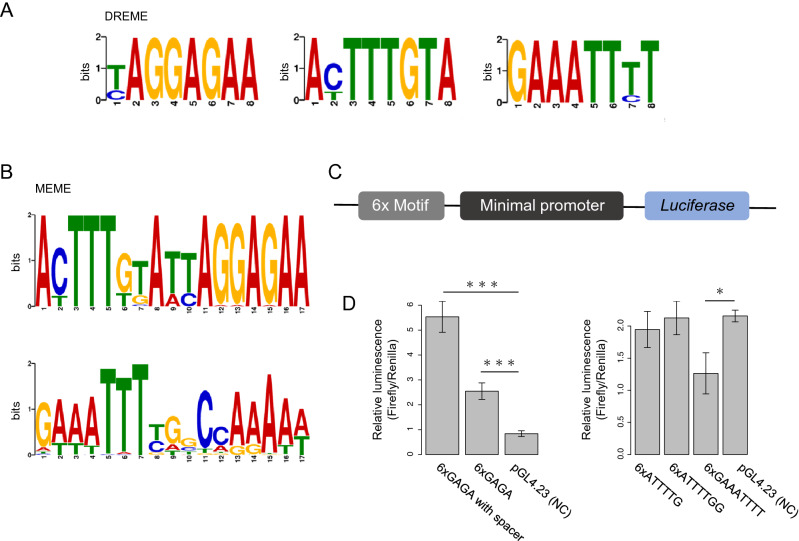
Mblk-1-binding motif analysis and luciferase assay. (**A**) Three Mblk-1-binding motifs identified with DREME. (**B**) Two Mblk-1-binding motifs identified with MEME. (**C**) pGL4.23 reporter vector construction used for the luciferase assay. (**D**) The results of the luciferase assay using reporter vectors containing motif 1: 6xGAGA or 6xGAGA with 4-bp spacer sequence (left panel) and motif 2: 6xGAAATTTT or motif 3: 6xATTTTGG sequence (right panel). Data represent means ± SD (n = 3). Significant differences are indicated by asterisks (*p < 0.05, **p < 0.01, ***p < 0.005, Tukey–Kramer test).

To search for Mblk-1-binding motifs, we next performed motif analysis using sequences corresponding to the Mblk-1-binding regions and MEME-ChIP^34^. Three consensus sequences were identified by DREME, which discovers short and ungapped motifs: GA-rich sequence (GAGA or GAG, hereafter termed a GAGA motif), GAAATTTT, and AT(C)TTTGTA (Fig. 3A). A combination of these motifs was also found by MEME, which discovers relatively long motifs (Fig. 3B). In a previous study, we identified an Mblk-1 preferred binding sequence named MBE (Mblk-1-binding element, 5′-AGGTAGAGATCGATCGATAGGG-3′) by using an in vitro binding site selection method^17^. The motif we found in this study is consistent with MBE, because MBE has a GAGA sequence. In addition, the ATTTTC(T)GG motif is reported as a binding motif of *Dm*E93^44^, and it is also reported that *Bombyx mori* E93 (*Bm*E93) binds to GAGA-containing motifs^45^. These previous findings support the quality of our data and suggest that Mblk-1-binding motifs are conserved in insects.

We next performed a luciferase assay to examine whether Mblk-1 recognizes these 3 binding motifs and affects the transcription of a reporter gene. Human HEK293T cells were transfected with an Mblk-1 expression vector; a *Renilla* luciferase reporter vector; and 5 types of pGL4.23 firefly luciferase reporter vectors, each of which contains 6xGAGA, 6xGAGA with 4-bp spacer sequences, 6xATTTTG, 6xGAAATTTT, or 6xATTTTGG upstream of the minimal promoter, respectively (Fig. 3C). Normalized firefly luciferase activity increased about 3.0-fold and 6.6-fold when co-transfected with pGL4.23 vectors containing 6xGAGA or 6xGAGA with 4-bp spacer sequences, respectively, compared with that when co-transfected with a pGL4.23 vector containing no binding motifs (Fig. 3D). This finding indicates that Mblk-1 recognizes GAGA motifs and upregulates the transcription of genes with these motifs. Normalized firefly luciferase activity, however, significantly decreased by approximately 60% when co-transfected with pGL4.23 vectors containing 6xGAAATTTT compared with that when co-transfected with a pGL4.23 vector containing no binding motifs (Fig. 3D). This finding suggests that Mblk-1 recognizes GAAATTTT motifs to downregulate the transcription of genes having this motif in contrast to those having GAGA motifs. In addition, no significant change in firefly luciferase activity was observed when co-transfected with a pGL4.23 vector containing 6xATTTTG or 6xATTTTGG (Fig. 3D), although the absence of an effect on the luciferase activity does not necessarily exclude the possibility that Mblk-1 binds to these motifs. Taken together, our results indicated that Mblk-1 affects gene transcription through GAGA or GAAATTTT motifs.

### Identification of genes upregulated in lKCs as candidates for Mblk-1 target genes

**Figure 4:**
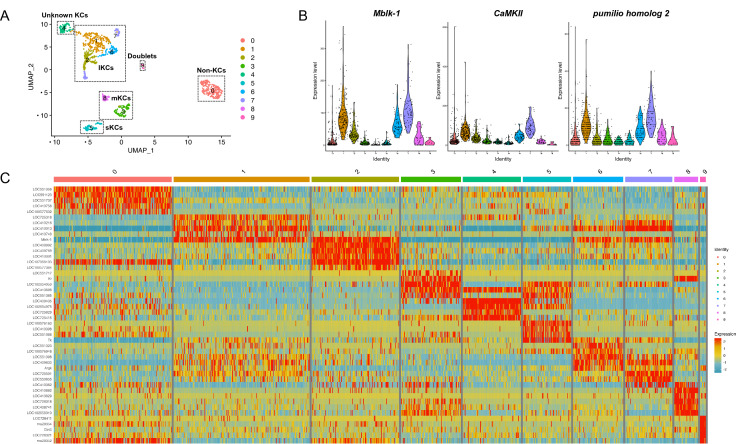

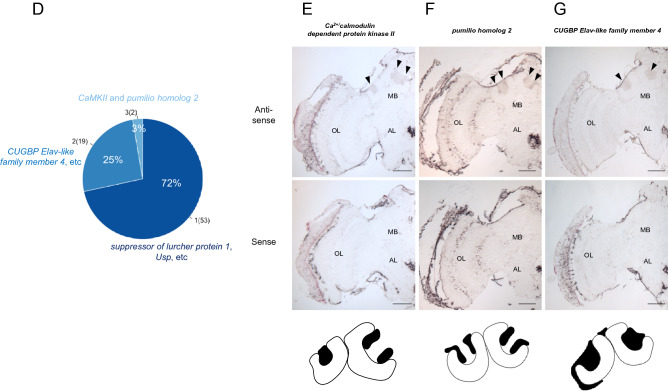
Single-cell RNA-seq data analysis using adult MBs and identification of Mblk-1 target gene candidates preferentially expressed in lKCs. (**A**) UMAP visualization of single-cell RNA-seq data obtained from adult honey bee MBs by Traniello et al.^36^. Clusters 1, 2, 6, and 7 correspond to lKCs. (**B**) Violin plots of gene expression: *Mblk-1*, *CaMKII*, and *pumilio homolog 2*. (**C**) Heatmap of expression of the top 5 cluster marker genes in each cluster. Positive values (shown in red or yellow) indicate that the gene is more highly expressed in the cluster indicated than in other clusters. (**D**) Ratios of genes suggested to be marker genes for 3 of the 4 clusters that correspond to lKCs (light blue), those for 2 clusters (blue), and those for 1 cluster (dark blue), respectively. Numbers in parentheses represent the number of Mblk-1 target gene candidates. (**E**–**G**) In situ hybridization results for the 3 marker genes. (**E**) *Ca*^*2*+^*/calmodulin-dependent protein kinase II* (**F**) *pumilio homolog 2* (**G**) *CUGBP Elav-like family member 4*. The upper panels show results using anti-sense RNA probes, whereas the lower panels show results using sense RNA probes. Schematic drawing of the expression pattern of each gene in the MB is shown at the bottom. *MB,* mushroom bodies; *OL,* optic lobes; *AL,* antennal lobes; *lKCs,* large-type KCs. Scale bar: 250 μm.

Given that Mblk-1 upregulates its target genes in a lKC-preferential manner in the honey bee MBs, Mblk-1 target gene candidates are expected to be expressed preferentially in lKCs. To find genes expressed preferentially in lKCs among the identified Mblk-1 target gene candidates, we used recently reported honey bee MB single-cell RNA-seq data^36^. When the MB cells were classified into 10 clusters (Fig. 4A), high expression of *PLCe* (LOC408804) was observed in clusters 1–8, but not in clusters 0 or 9 (Fig. S2A). We considered clusters 1–8 with high *PLCe* expression to be KCs, because we previously reported high expression of this gene in all KCs (the entire MBs)^39^. Of these 8 clusters, preferential *Mblk-1* expression was observed in clusters 1, 2, 6, and 7 (Fig. 4B). In addition to *Mblk-1*, genes indicated as preferentially expressed in lKCs by in situ hybridization, such as *disks large homolog 5* (LOC410178)^39^ and *Ryr* (LOC408680)^46^, were highly expressed at least in 1 of the 4 clusters (clusters 1, 2, 6, and/or 7) (Fig. S2B,C and Supplementary Data 4). Therefore, we regarded these 4 clusters as lKCs. Preferential expression of *middle-type Kenyon cell-preferential arrestin-related protein* (LOC725542), which is reported to be expressed in mKCs^47^, was observed in clusters 0, 3, and 8 (Fig. S2D and Supplementary Data 4). This finding suggests that clusters 3 and 8 corresponded to mKCs. Although the expression level of *PLCe* in cluster 0 was lower than that in other clusters, the expression level of the pan-neuronal marker *ELAV-like protein 2* (LOC410689) was high (Fig. S2E), suggesting that cells in this cluster are non-KCs (possibly optic lobe neurons) that contaminated the MB samples. In cluster 4, the most preferentially expressed gene was *POU domain, class 6, transcription factor 2* (LOC408435) (Fig. 4C and Supplementary Data 4), but no known KC-subtype marker genes were suggested and therefore the known KCs to which this cluster corresponds remain unclear. Among the 8 clusters corresponding to KCs, *Tk* (*tachykinin*) appeared to be preferentially expressed in cluster 5 (Fig. 4C). This gene is expressed in both sKCs and lKCs, with greater expression in sKCs than in lKCs^48^. In the violin plot, *Tk* expression was highest in cluster 5 (Fig. S2F), suggesting that cluster 5 corresponds to sKCs. Genes specifically expressed in Class II KCs have not yet been identified by in situ hybridization analyses^12^, making it difficult to identify which clusters correspond to Class II KCs. Cluster 9 likely comprises doublet cells, because more than half of the cells in this cluster were determined to be doublets by DoubletFinder (Fig. S3).

We identified 75 genes as possible Mblk-1 target genes, including *Mblk-1* itself, with higher expression in at least one of clusters 1, 2, 6, and 7 than in the other clusters (Supplementary Data 5). Of these 75 genes, 2 genes (*CaMKII* and *pumilio homolog 2*) were suggested to be marker genes for 3 of the 4 clusters comprising lKCs (Fig. 4D and Supplementary Data 4); 19 genes, including *CUGBP Elav-like family member 4*, were marker genes for 2 of the 4 clusters; and 53 genes, including *suppressor of lurcher protein 1* and *Usp*, were marker genes for 1 of the 4 clusters (Fig. 4D). In situ hybridization analysis of these genes was performed to examine whether they are preferentially expressed in lKCs. *CaMKII* was confirmed to be expressed preferentially in lKCs (Fig. 4E), consistent with a previous report^13^. In addition, we found preferential expression of *pumilio homolog 2* in lKCs (Fig. 4F). *CUGBP Elav-like family member 4* was expressed in the whole MBs, but not in other brain regions (Fig. 4G), consistent with the result of scRNA-seq analysis showing that *CUGBP Elav-like family member 4* was also a marker gene for clusters other than the 4 clusters, which correspond to lKCs (Supplementary Data 4). Schematic diagrams of the peak signals located around *CUGBP Elav-like family member 4* and *pumilio homolog 2* are shown in Fig. S4.

### Comparison of Mblk-1 target gene candidate profiles between pupal and adult worker honey bee brains

**Figure 5:**
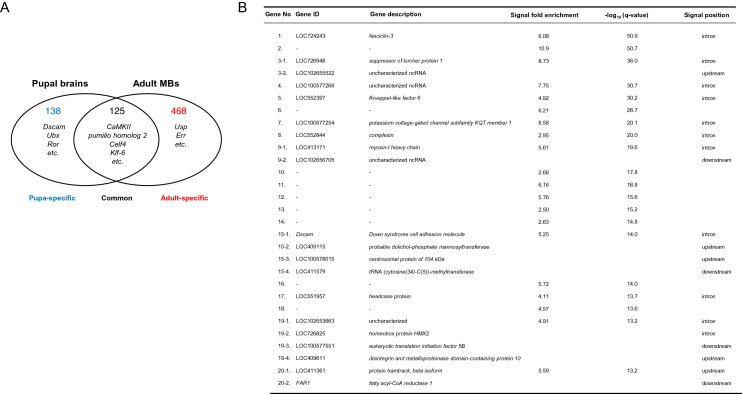
Identification of Mblk-1 target gene candidates in pupal brains. (**A**) Venn diagram showing the numbers of Mblk-1 target gene candidates identified as pupal- or adult-specific and common target genes. (**B**) List of the top 20 ChIP-seq peak signals identified from pupal worker brain homogenates and Mblk-1 target gene candidates located within ± 10 kb of the corresponding signals. The Gene ID, gene description, signal fold enrichment, − log_10_ (q-value), and signal position relative to the genes are shown for each ChIP-seq peak signal. Dashes indicate the absence of genes located within ± 10 kb of the corresponding peak signal. Note that side numbers (e.g., 3-1 and 3-2) indicate that multiple genes are located within ± 10 kb of the corresponding peak (e.g., peak 3).

Mblk-1 expression level is higher in pupal brains than adult brains^18^. In addition, *Dm*E93 is expressed transiently in pupal MB neuroblasts to activate autophagy during metamorphosis^24^. Thus, we next examined whether Mblk-1 has distinct target genes between adult and pupal worker brains in the honey bee. To this end, we performed ChIP-seq analysis using pupal worker brain homogenates and the anti-Mblk-1 antibody. A total of 263 genes were identified as pupal Mblk-1 target gene candidates, for which Mblk-1-binding signals are located within 10 kb upstream or downstream of the corresponding genes when using a peak calling q-value threshold of 0.005 and the input DNA sample as a control. Approximately half of the pupal Mblk-1 target gene candidates (138/263, 52%) were detected specifically in the ChIP-seq analysis using pupal brains (Fig. 5A, Supplementary Data 2 and 3). The Mblk-1-binding signals with the 20 lowest q-values (top 20 signals) and genes that have these signals within 10 kb upstream or downstream are shown in Fig. 5B. Among the 20 pupal target gene candidates shown in Fig. 5B, 8 genes were pupal-specific (8/20, 40%). For almost half of the 20 signals (9/20, 45%), no genes were localized within 10 kb upstream or downstream of the signals. For example, a neural developmental gene, *Down syndrome cell adhesion molecule* (*Dscam*) shown in Fig. 5B was not present among the adult target gene candidates, suggesting that this gene is a pupal-specific target. The peak signal was found in the first intron of *Dscam*, and only the GAGA motif was found in the Mblk-1-binding region. In addition to *Dscam*, several developmental genes, including *Ultrabithorax* and *Tyrosine-protein kinase transmembrane receptor Ror*, were specifically detected in the pupal analysis (Supplementary Data 2).

We performed GO enrichment analysis for adult-specific, pupal-specific, or common target gene candidates by Metascape^33^. The target gene candidates used for the GO enrichment analysis are listed in Supplementary Data 6. In our analysis of the adult-specific target gene candidates, generation of neurons (GO:0048699) and open tracheal system development (GO:0007424) were listed as the top 2 (Fig. S5). On the other hand, in our analysis of the pupal-specific target gene candidates, compound eye development (GO:0048749) and urogenital system development (GO:0001655) were suggested as the top 2 (Fig. S6). We also performed GO enrichment analysis using the common target gene candidates and locomotion (GO:0040011) was suggested as the top GO term (Fig. S7).

### Self-regulation of Mblk-1 expression in honey bee MBs

**Figure 6:**
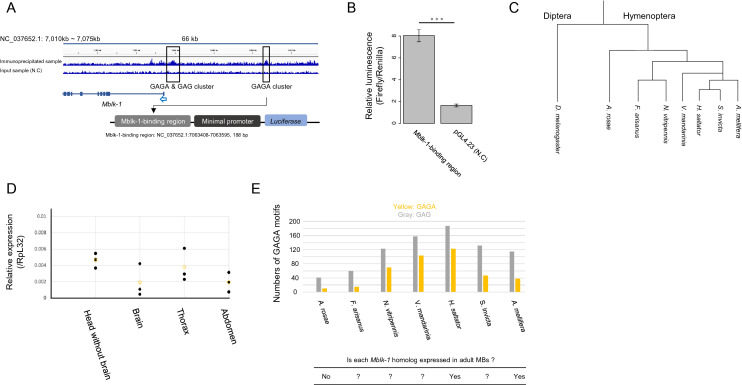
Mblk-1 upregulates its own expression via GAGA motif-containing regions. (**A**) Two ChIP-seq peak signals were identified upstream of the *Mblk-1* gene (outlined in black). Construction of the pGL4.23 reporter vector used in the luciferase assay is indicated below the *Mblk-1* gene structure. The transcription start site of the *Mblk-1* gene is indicated by a blue arrow. (**B**) Results of the luciferase assay using a reporter vector containing the Mblk-1-binding region sequence and a control vector. Data represent means ± SD (n = 4). Significant difference is indicated by 3 asterisks (p < 0.005, Student’s *t* test). (**C**) Phylogenetic tree showing the relationship among hymenopteran species and *Drosophila melanogaster*. (**D**) Comparison of the relative expression of the *Athalia rosae Mblk-1* homolog with respect to *RpL32* among various adult body parts (head without brain, brain, thorax, and abdomen, n = 3). Black circles represent values from 3 experiments and yellow circles represent their average values. Because expression of the *Mblk-1* homolog in brain samples was below the detection threshold, brain Cp values were set to 40 in a total of 45 PCR cycles and the relative expression levels were calculated. (**E**) Relationship between the number of GAGA and GAG sequences localized within 2 kb upstream of *Mblk-1* homologs and expression of *Mblk-1* homologs in adult MBs of various hymenopteran insect species. Yellow and gray bars indicate the number of GAGA and GAG sequences, respectively. Table below shows whether *Mblk-1* homolog is expressed in the adult MBs in each species.

Interestingly, two distinct peak signals were found 2 kb and 25 kb upstream of the *Mblk-1* gene, respectively (Fig. 6A). All these signals contained GAGA motifs, which induced the expression of the downstream *luciferase* gene in the reporter assay (Fig. 3D). From this result, we expected that Mblk-1 induces its own expression via these GAGA motifs. To confirm this, we performed a luciferase assay with a pGL4.23 firefly luciferase reporter vector that contains the sequence corresponding to the peak signal located 25 kb upstream of *Mblk-1* gene (Fig. 6A). We observed an approximately 4.9-fold increase in firefly luciferase activity when using the pGL4.23 vector containing the peak signal sequence compared with that when using the pGL4.23 vector containing no binding motifs (Fig. 6B). Therefore, Mblk-1 is suggested to recognize this GAGA-containing region as an enhancer to upregulate its own expression. It is possible that once *Mblk-1* is expressed, it can upregulate its own expression, which leads to its constitutive expression in adult honey bee lKCs.

### Expression analysis of *Mblk-1* homolog in the sawfly

In the honey bee, *Mblk-1* is almost selectively expressed in the brain among various adult tissues^18^. In addition, in the ant *Harpegnathos saltator* workers and gamergates, the expression of the *Mblk-1* homolog in the brain is observed^49^. We next tested whether the constitutive expression of *Mblk-1* homologs in the adult brain is conserved even in other hymenopteran insect species (Fig. 6C). We performed qRT-PCR analysis to examine the expression of the *Mblk-1* homolog (LOC105691346) in various adult body parts of the sawfly (*Athalia rosae*), which is a solitary, phytophagous, and primitive hymenopteran insect species^43^. Although significant expression of the sawfly *Mblk-1* homolog was detected in the head without the brain, thorax, and abdomen, the expression in the brain was below the detection threshold (crossing point Cp: 40) (Table S2). The relative expression levels of the *Mblk-1* homolog did not differ significantly among body parts when the brain Cp values were set to 40 (Fig. 6D). These results indicate that the expression level of *Mblk-1* homolog is very low in the sawfly brain, and that brain-specific expression, which is observed in the honey bee, is not conserved in the sawfly.

### Comparative analysis of the upstream sequences of *Mblk-1* homologs

Finally, we investigated how accumulation of the GAGA motif (GAGA and GAG) related to the expression of *Mblk-1* homologs in the brains of hymenopteran insects. We counted the number of GAGA motifs located within 2 kb and 10 kb from the transcription start site of each *Mblk-1* homolog (Fig. 6E and Fig. S8). In all 4 species of Aculeata, the number of GAG sequences within 2 kb upstream was greater than 100, and the number of GAGA sequences was greater than 30. On the other hand, in *Athalia rosae*, in which no *Mblk-1* homolog expression was observed in adult brains, the number of GAG sequences was 41. In addition, to determine the evolutional stage in hymenopteran insects at which the accumulation of GAGA motifs was acquired, we investigated the numbers of GAGA motifs in the 2 parasitoid wasps, *Nasonia vitripennis* and *Fopius arisanus*. The numbers of GAGA motifs in *Nasonia vitripennis* were comparable to those in Aculeata, while the numbers in *Fopius arisanus* were closer to those in *Athalia rosae*. The same trend was observed in the numbers of GAGA motifs within upstream of 10 kb (Fig. S8).

## Discussion

### Mblk-1 targets genes related to synaptic plasticity and ecdysone-signaling in adult honey bee MBs

In the present study, we newly identified genes related to neural function, such as *CaMKII* and *pumilio homolog 2* as Mblk-1 target gene candidates in the worker honey bee MBs by ChIP-seq analyses. *CaMKII* is involved in synaptic functions^25–27^. In addition, *Drosophila melanogaster pumilio* has a role in synaptic plasticity and learning ability^50^. Therefore, it is likely that Mblk-1 is involved in neural function, such as learning and memory abilities through transcriptional regulation of these synaptic plasticity-related genes.

Several ecdysone-related genes such as *Usp* were also identified as Mblk-1 target gene candidates. In forager bees (> 21 days), the expression of *Usp* is stronger in lKCs than in the inner compact KCs, which correspond to mKCs and sKCs^51^. It is likely that *Usp* is regulated differently between lKCs and inner compact KCs, and its expression in lKCs is controlled by *Mblk-1*. It is plausible that *Usp* has some neural functions that differ from its developmental functions like *Mblk-1*. This is consistent with reports that ecdysone and ecdysone-related genes are involved in neural functions, including learning and memory abilities, in *Drosophila melanogaster*^52^.

### The honeybee Mblk-1 has three DNA binding motifs that are shared with *Dm*E93 or *Bm*E93

We identified three Mblk-1-binding motifs (GAGA, GAAATTTT, and AT(C)TTTGTA; Fig. 3A,B). The luciferase assay suggested that the GAGA and GAAATTTT motifs have different functions in transcriptional regulation by Mblk-1. GAGA and ATTTTGG motifs are suggested to be binding motifs for *Bm*E93 and *Dm*E93, respectively^44,45^, indicating that these two Mblk-1-binding motifs are conserved in insects. To the best of our knowledge, however, only AWTTTYGG and HTTTBGG, both of which are like ATTTTGG, are reported as *Dm*E93-binding motifs in Fly Factor Survey and previous research using ChIP-seq analysis^44^. It is thus likely that Mblk-1 possesses a binding motif (the GAGA motif) that is not observed in *Dm*E93. In the luciferase assay experiments, the expression of a reporter gene was upregulated when using the reporter vector containing the 6xGAGA sequence (Fig. 3D). Therefore, it is probable that Mblk-1 recognizes the GAGA motifs and upregulates the expression of genes around them in lKCs.

### Distinct functions of Mblk-1 between pupal and adult brains

We identified pupal-specific Mblk-1 target gene candidates (Supplementary Data 2), which include some neural developmental genes. Approximately half of the target gene candidates for pupal brains were not identified as Mblk-1 target gene candidates for adult MBs (Fig. 5A), suggesting that Mblk-1 alters its role by changing its target genes as honey bees develop from pupae to adults. There are two possible explanations for this change. One is that target genes of Mblk-1 that functions in regions other than MBs, such as OLs and ALs where Mblk-1 is expressed in early pupal stage^39^, were included since we used pupal whole brains for the ChIP-seq analysis. The other possible explanation is that Mblk-1 regulates different genes when it is phosphorylated. Mblk-1 is phosphorylated by mitogen-activated protein kinase^35^ in a pupae-specific manner^18^. The anti-Mblk-1 antibody was designed to recognize both non-phosphorylated and phosphorylated Mblk-1^18^. It is thus possible that Mblk-1 changes its binding regions and target genes depending on phosphorylation.

The GO enrichment analysis of adult-specific target gene candidates suggested GO terms related to development, such as generation of neurons (GO:0048699) and open tracheal system development (GO:0007424) (Fig. S5), although the genes used in the analysis were obtained from the ChIP-seq experiment using adult MBs. This finding indicates that Mblk-1 in adult MBs regulates the expression of genes involved in development as well as those related to synaptic plasticity. It is likely that the functions of these developmental genes in adult MBs are different from the ones in developing pupae. Similarly, in the GO enrichment analysis of the pupal-specific target gene candidates, GO terms related to development, such as compound eye development (GO:0048749) and urogenital system development (GO:0001655), were suggested (Fig. S6). Because the genes used in the analysis were obtained from the ChIP-seq experiment using pupal brains, it is reasonable to assume that these genes are involved in neural development. In conclusion from the GO enrichment analyses, many of the Mblk-1 target gene candidates are related to development as expected from their conventional role, but some have neural functions in adult MBs.

### Self-regulation: a possible model for constitutive expression of *Mblk-1* in adult honey bee MBs

Two distinct peak signals were found upstream of the *Mblk-1* gene, and the GAGA motif (GAGA or GAG) is enriched in those signal regions. The results of the luciferase assay indicated that the DNA sequence corresponding to one of the peak signals was recognized by Mblk-1 and the expression of its downstream reporter gene was upregulated (Fig. 6B). Generally, in insect metamorphosis, *E93* is transiently induced by ecdysone and ecdysone receptors^20^. On the other hand, Mblk-1 is constitutively expressed in honey bee lKCs^18^, whereas *ecdysone receptor* (*EcR*) is mainly expressed in sKCs in the honey bee MBs^53^. Therefore, together with the result of the luciferase assay, it is likely that the constitutive expression of Mblk-1 in lKCs is, at least partially, accounted for by self-regulation instead of ecdysone-dependent regulation. *Mblk-1* expression seems to be initiated in the pupal stage, because its expression is observed in early pupae (P2), but not in the larval brain^39^. It is likely that *Mblk-1* is first induced in the pupal brain under the control of ecdysone-EcR-Usp ternary complex, as suggested by earlier reports^20,21^. We hypothesize that Mblk-1, once activated, induces its own expression autonomously by self-regulation through the pupal to adult stages.

The above luciferase assay finding also suggests that the accumulation of the GAGA motif sequences upstream of *Mblk-1* is important in the self-induction of *Mblk-1*. The numbers of the GAGA motif sequences in species in which the constitutive expression of *Mblk-1* homologs in adult brains are observed (*Apis mellifera* and *Harpegnathos saltator*^49^) were much greater than those in *Athalia rosae*. Consistent with this, the qRT-PCR analysis using sawfly body parts indicated that the *Mblk-1* homolog is not selectively expressed in the brains of adult sawflies (Fig. 6D). For *Nasonia vitripennis*, the GAGA motifs are accumulated upstream of the *Mblk-1* homolog, although we have not analyzed whether their *Mblk-1* homologs are preferentially expressed in the adult brain. If the GAGA motif accumulation and the constitutive expression of *Mblk-1* homologs in brains has been acquired in parasitoid wasps, the role of *Mblk-1* homologs related to neural functions, including learning and memory abilities, might have been a platform for the regulation of parasitic behaviors and prerequisite for further advanced behaviors of aculeate species.

Recently, Davie et al*.*^54^ reported the results of scRNA-seq using the adult *Drosophila* brain. When we investigated the expression of *DmE93* using the data by Davie et al.^54^, we found that *DmE93* was identified as a cluster marker in 25 among 87 clusters, and 3 of the former 25 clusters correspond to KCs. Therefore, unlike *Mblk-1*, *DmE93* is expressed in broad brain regions, including the whole MBs, in the fly brain. In addition, from regulons suggested in the *SCope*, *DmE93* is not likely to target *CaMKII* or *pum* in the fly brain. From the expression pattern of *DmE93*, it seems that the constitutive expression of *E93* in MBs itself is not directly related to the MB function specific to the honey bee. Our present findings suggest that both the autonomous and preferential expression of *Mblk-1* in lKCs and its altered binding-specificity are important for the function of *Mblk-1* in lKCs in honey bees.

In summary, although Mblk-1/E93 is known as a transcription factor required for metamorphosis in insects^21–24^, the findings of the present study indicate that the roles of Mblk-1 have expanded to regulate synaptic plasticity. By acquiring new expression mechanisms, binding motifs, and target genes during the evolution of hymenopteran insects, Mblk-1 may contribute to neural development in pupae and the advanced learning and memory abilities in adults. Our study provides an example of molecular evolution that could contribute to the acquisition of novel MB function in insects. Similar mechanisms may be at work in the brains of animals in general, and our findings could contribute to a better understanding of the evolution of brains and behaviors.

## Supplementary Information

Supplementary Information 1.

Supplementary Information 2.

Supplementary Information 3.

Supplementary Information 4.

Supplementary Information 5.

Supplementary Information 6.

Supplementary Information 7.

Supplementary Information 8.

## Data Availability

ChIP-seq data in this study were deposited in the GEO with the accession number GSE173409. The link for data access is as follows: https://www.ncbi.nlm.nih.gov/geo/query/acc.cgi?acc=GSE173409. We declare that all data supporting the findings of this study are available within the article and its supplementary information files.
