# Supplementary material for: Mblk-1/E93, an ecdysone related-transcription factor, targets synaptic plasticity-related genes in the honey bee mushroom bodies
Source: Sci Rep. 2022 Dec 9;12:21367. doi: 10.1038/s41598-022-23329-z (PMC9734179; doi:10.1038/s41598-022-23329-z)
Supplement: Supplementary file 7 — Supplementary Information 7. [file 41598_2022_23329_MOESM7_ESM.pptx]

## Slide 1
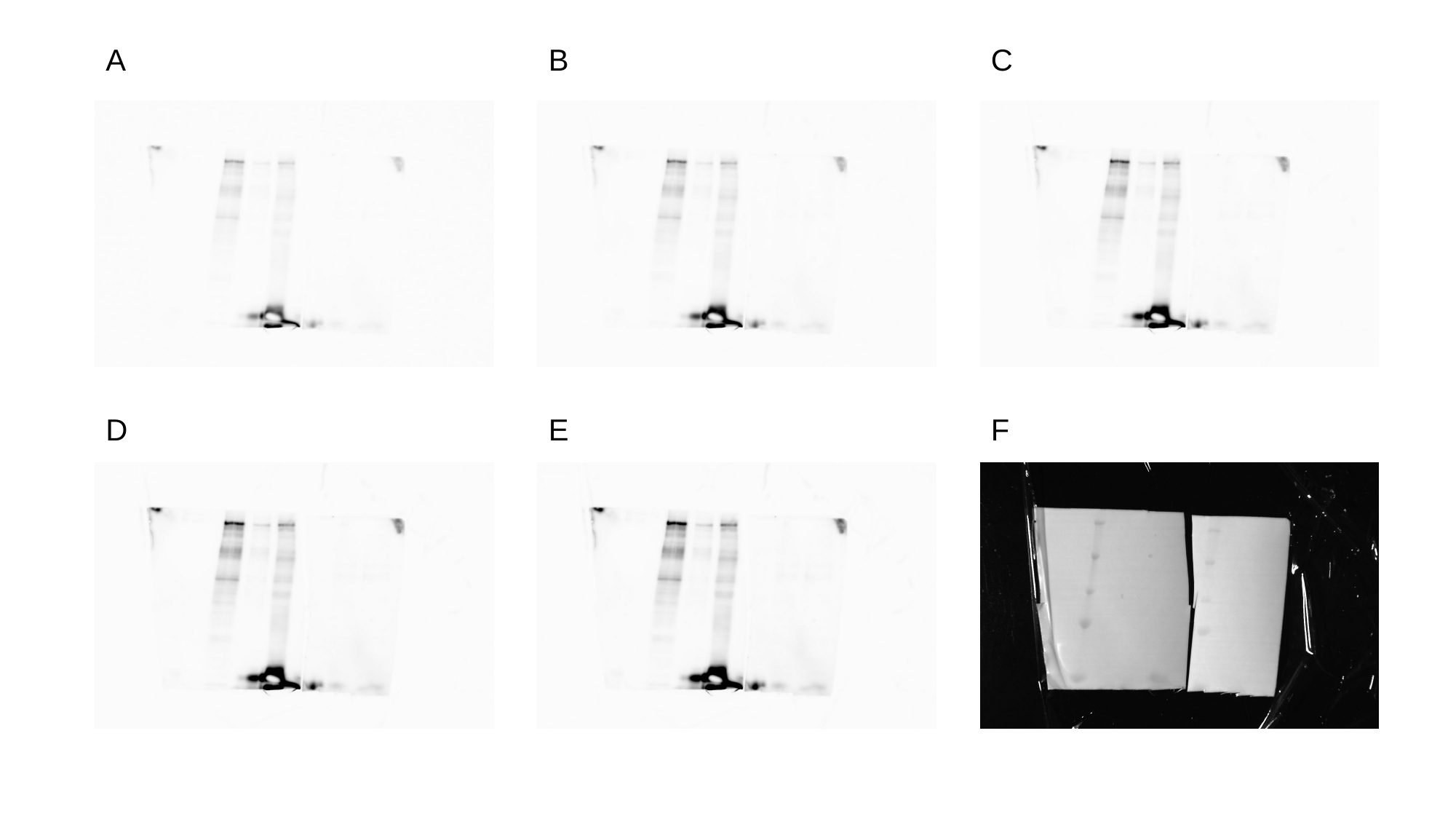

A
B
C
D
E
F

## Slide 2
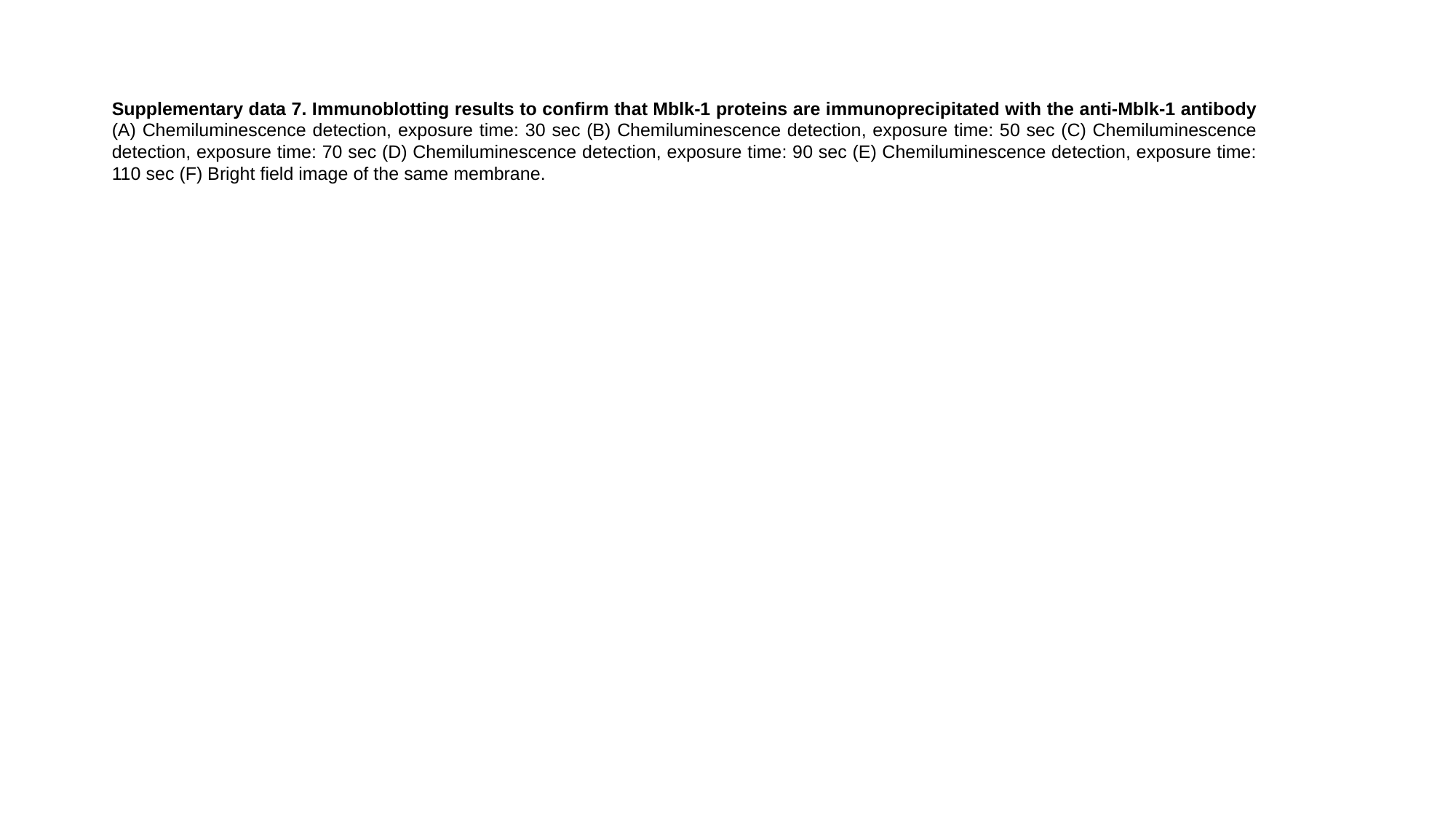

Supplementary data 7. Immunoblotting results to confirm that Mblk-1 proteins are immunoprecipitated with the anti-Mblk-1 antibody (A) Chemiluminescence detection, exposure time: 30 sec (B) Chemiluminescence detection, exposure time: 50 sec (C) Chemiluminescence detection, exposure time: 70 sec (D) Chemiluminescence detection, exposure time: 90 sec (E) Chemiluminescence detection, exposure time: 110 sec (F) Bright field image of the same membrane.
