# Supplementary material for: Mblk-1/E93, an ecdysone related-transcription factor, targets synaptic plasticity-related genes in the honey bee mushroom bodies
Source: Sci Rep. 2022 Dec 9;12:21367. doi: 10.1038/s41598-022-23329-z (PMC9734179; doi:10.1038/s41598-022-23329-z)
Supplement: Supplementary file 8 — Supplementary Information 8. [file 41598_2022_23329_MOESM8_ESM.pptx]

## Slide 1
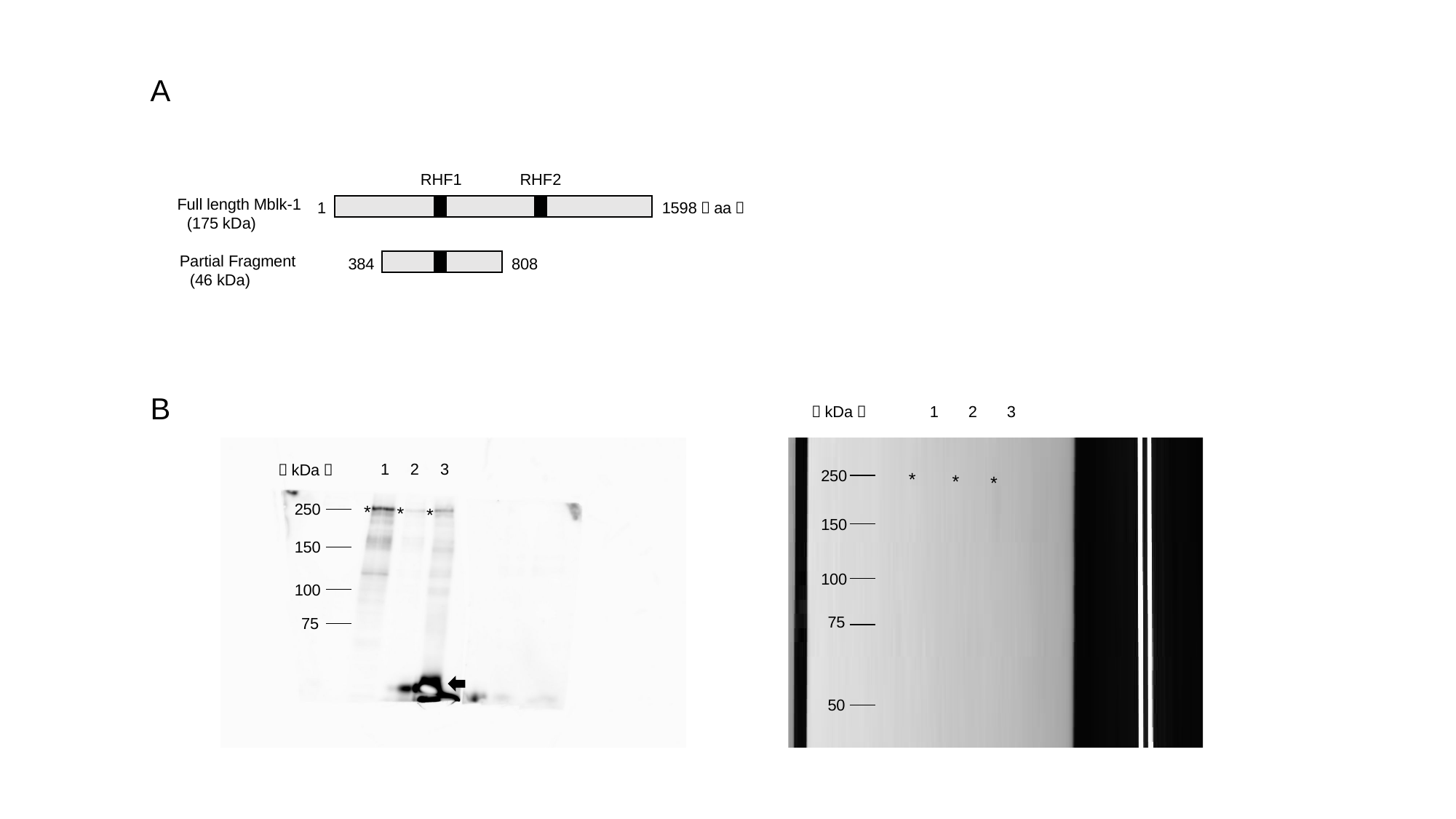

A
RHF1
RHF2
Full length Mblk-1
(175 kDa)
1
1598（aa）
Partial Fragment
(46 kDa)
384
808
B
（kDa）
1
2
3
1
2
3
（kDa）
250
*
*
*
250
*
*
*
150
150
100
100
75
75
50

## Slide 2
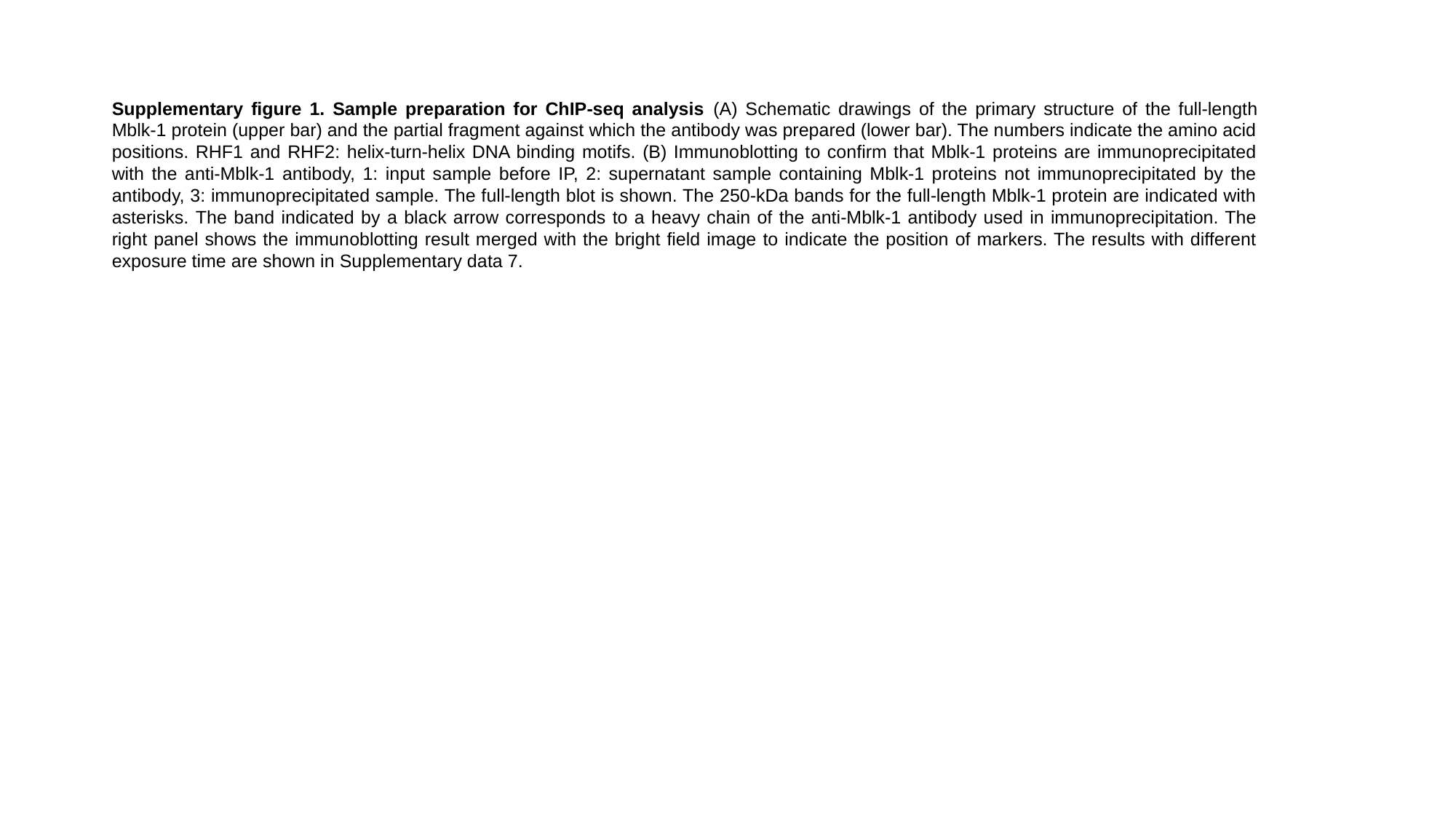

Supplementary figure 1. Sample preparation for ChIP-seq analysis (A) Schematic drawings of the primary structure of the full-length Mblk-1 protein (upper bar) and the partial fragment against which the antibody was prepared (lower bar). The numbers indicate the amino acid positions. RHF1 and RHF2: helix-turn-helix DNA binding motifs. (B) Immunoblotting to confirm that Mblk-1 proteins are immunoprecipitated with the anti-Mblk-1 antibody, 1: input sample before IP, 2: supernatant sample containing Mblk-1 proteins not immunoprecipitated by the antibody, 3: immunoprecipitated sample. The full-length blot is shown. The 250-kDa bands for the full-length Mblk-1 protein are indicated with asterisks. The band indicated by a black arrow corresponds to a heavy chain of the anti-Mblk-1 antibody used in immunoprecipitation. The right panel shows the immunoblotting result merged with the bright field image to indicate the position of markers. The results with different exposure time are shown in Supplementary data 7.

## Slide 3
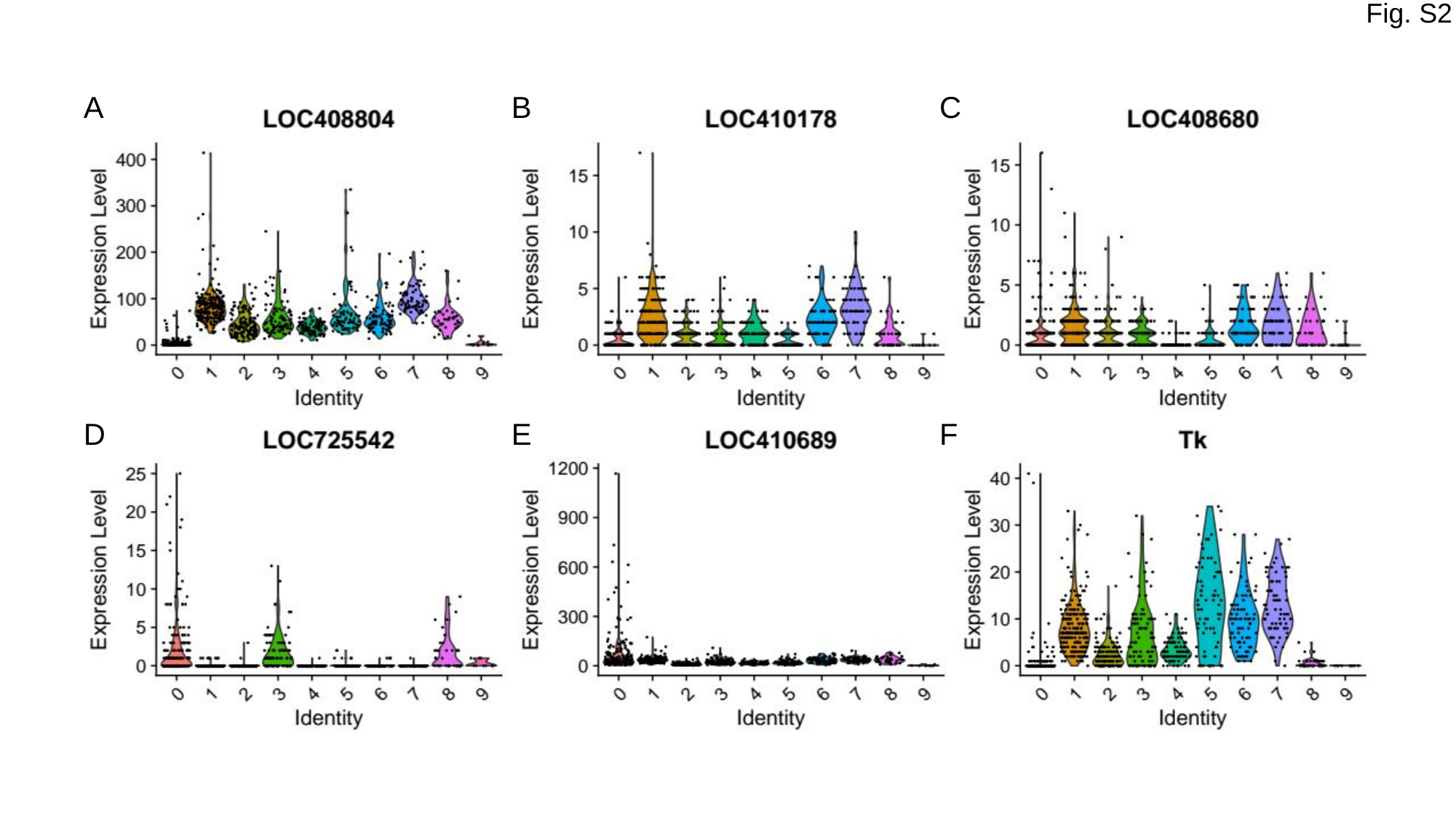

Fig. S2
A
B
C
D
E
F

## Slide 4
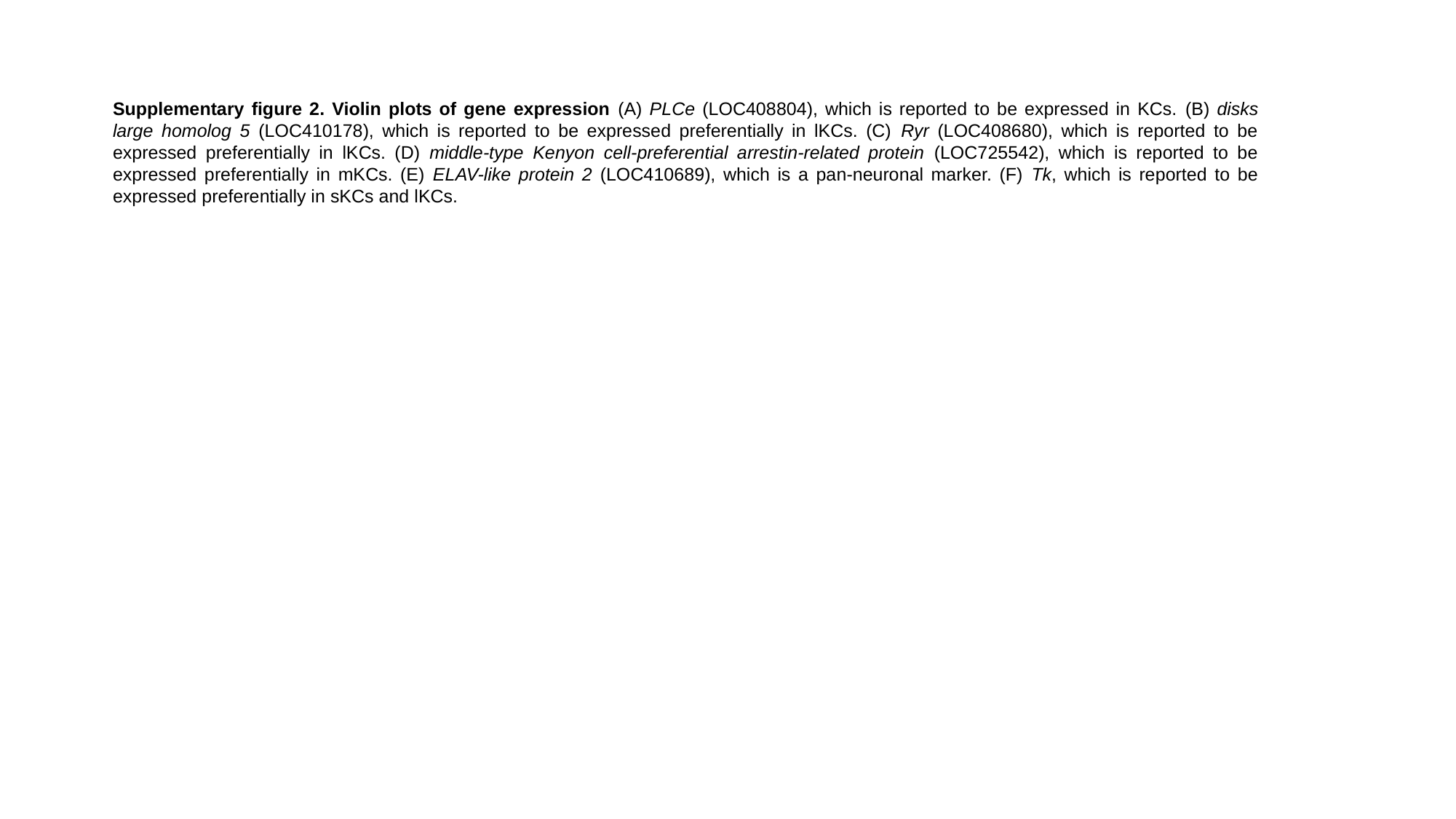

Supplementary figure 2. Violin plots of gene expression (A) PLCe (LOC408804), which is reported to be expressed in KCs. (B) disks large homolog 5 (LOC410178), which is reported to be expressed preferentially in lKCs. (C) Ryr (LOC408680), which is reported to be expressed preferentially in lKCs. (D) middle-type Kenyon cell-preferential arrestin-related protein (LOC725542), which is reported to be expressed preferentially in mKCs. (E) ELAV-like protein 2 (LOC410689), which is a pan-neuronal marker. (F) Tk, which is reported to be expressed preferentially in sKCs and lKCs.

## Slide 5
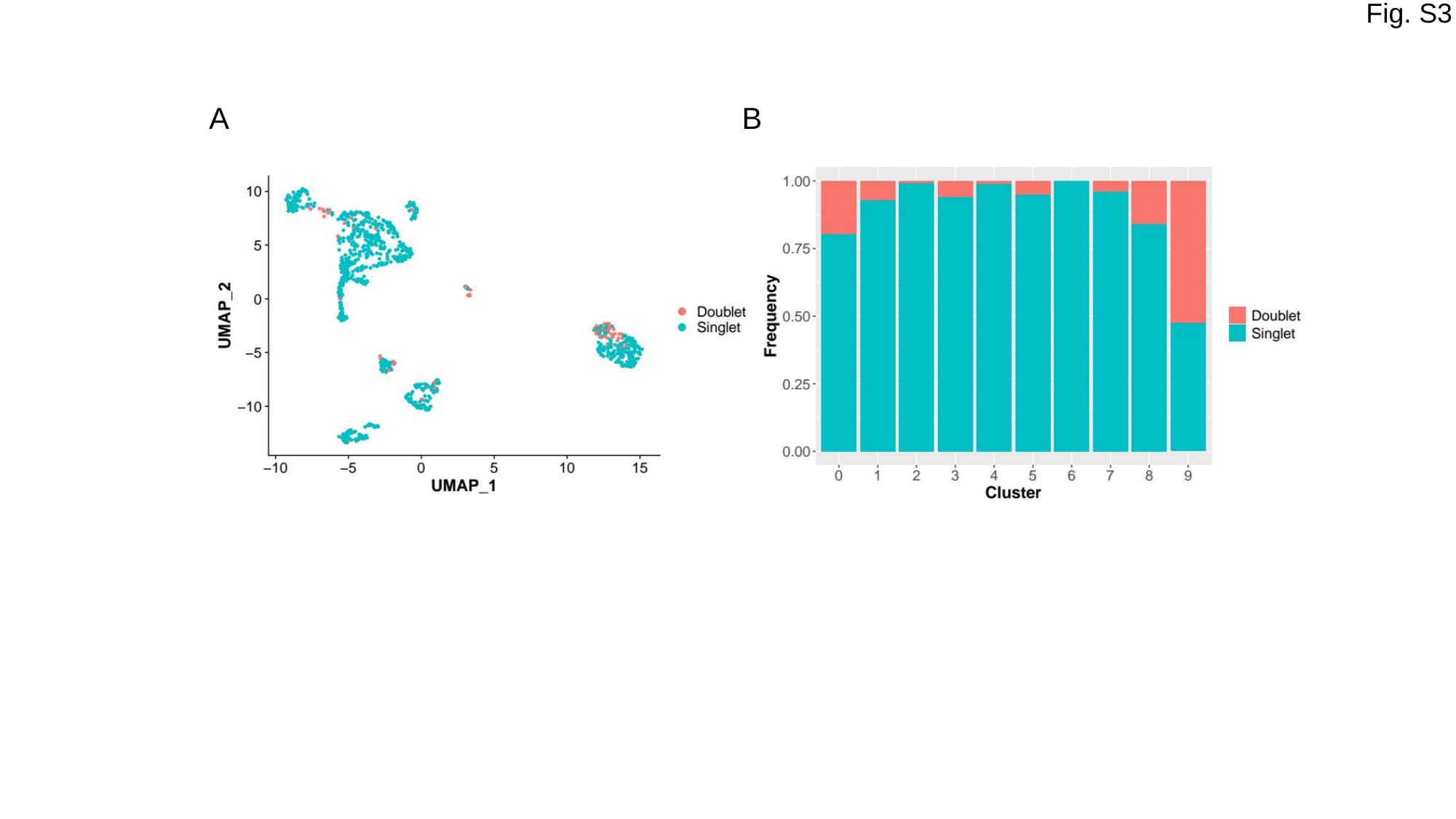

Fig. S3
A
B

## Slide 6
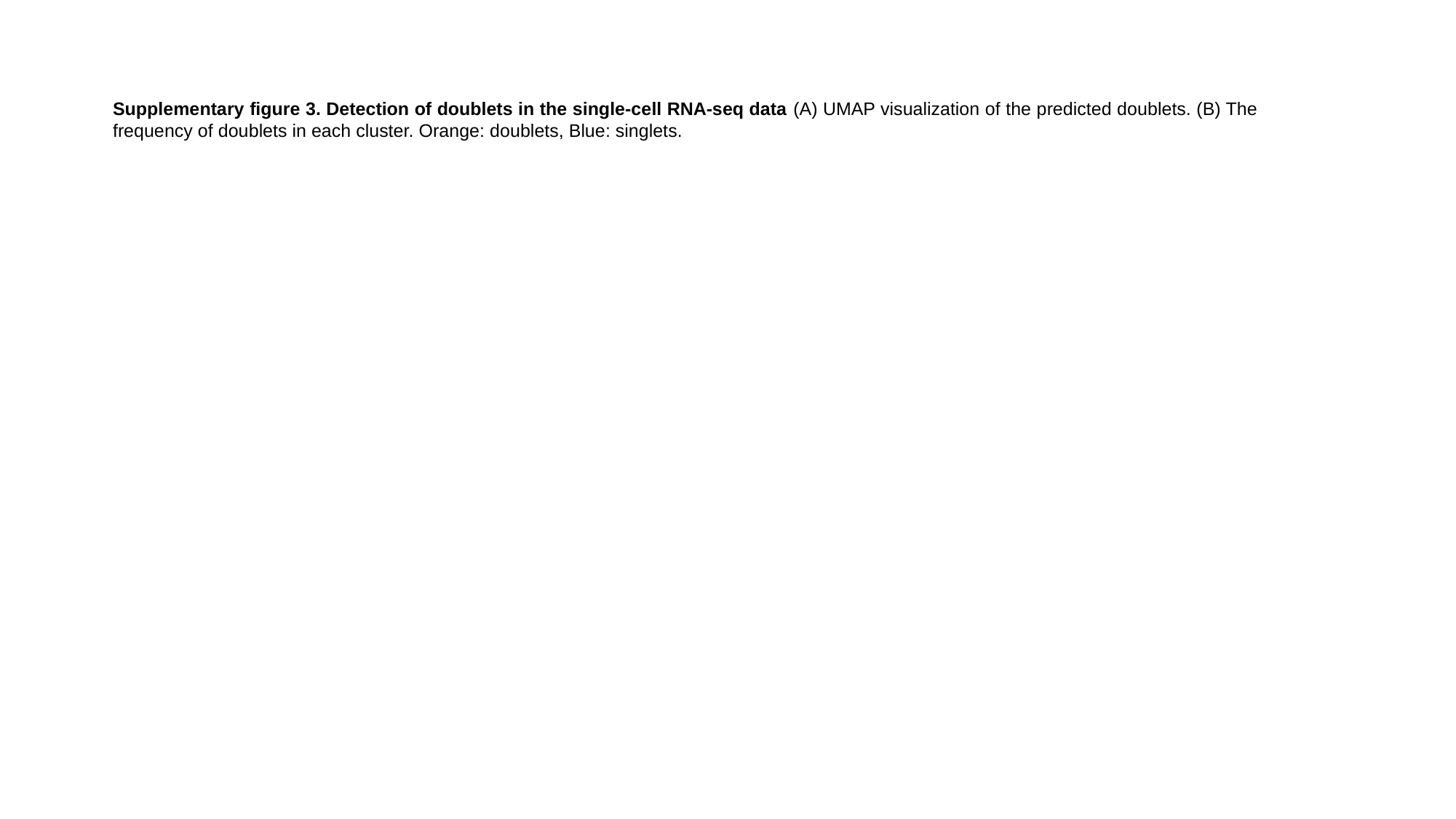

Supplementary figure 3. Detection of doublets in the single-cell RNA-seq data (A) UMAP visualization of the predicted doublets. (B) The frequency of doublets in each cluster. Orange: doublets, Blue: singlets.

## Slide 7
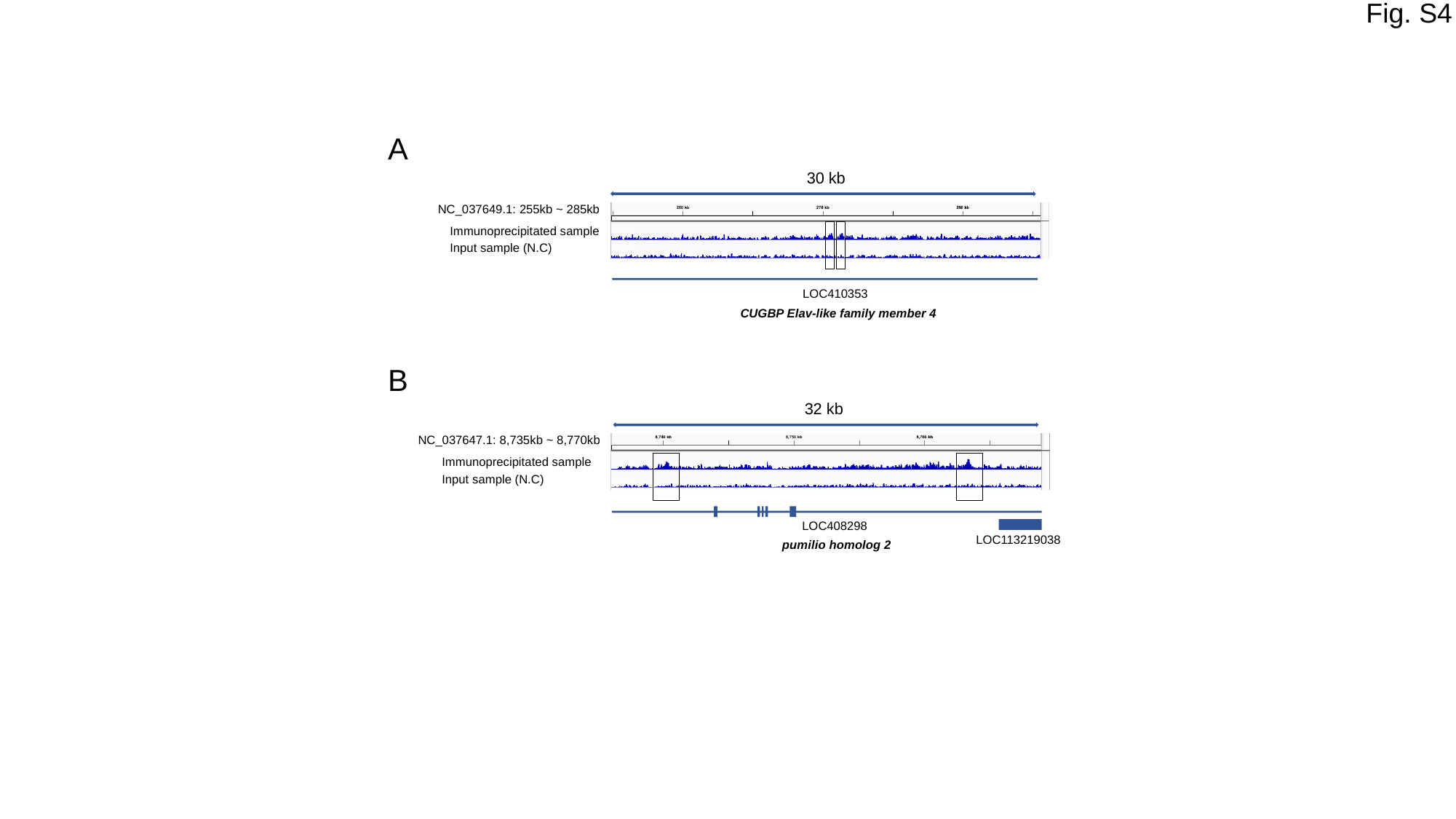

Fig. S4
A
30 kb
NC_037649.1: 255kb ~ 285kb
Immunoprecipitated sample
Input sample (N.C)
LOC410353
CUGBP Elav-like family member 4
B
32 kb
NC_037647.1: 8,735kb ~ 8,770kb
Immunoprecipitated sample
Input sample (N.C)
LOC408298
LOC113219038
pumilio homolog 2

## Slide 8
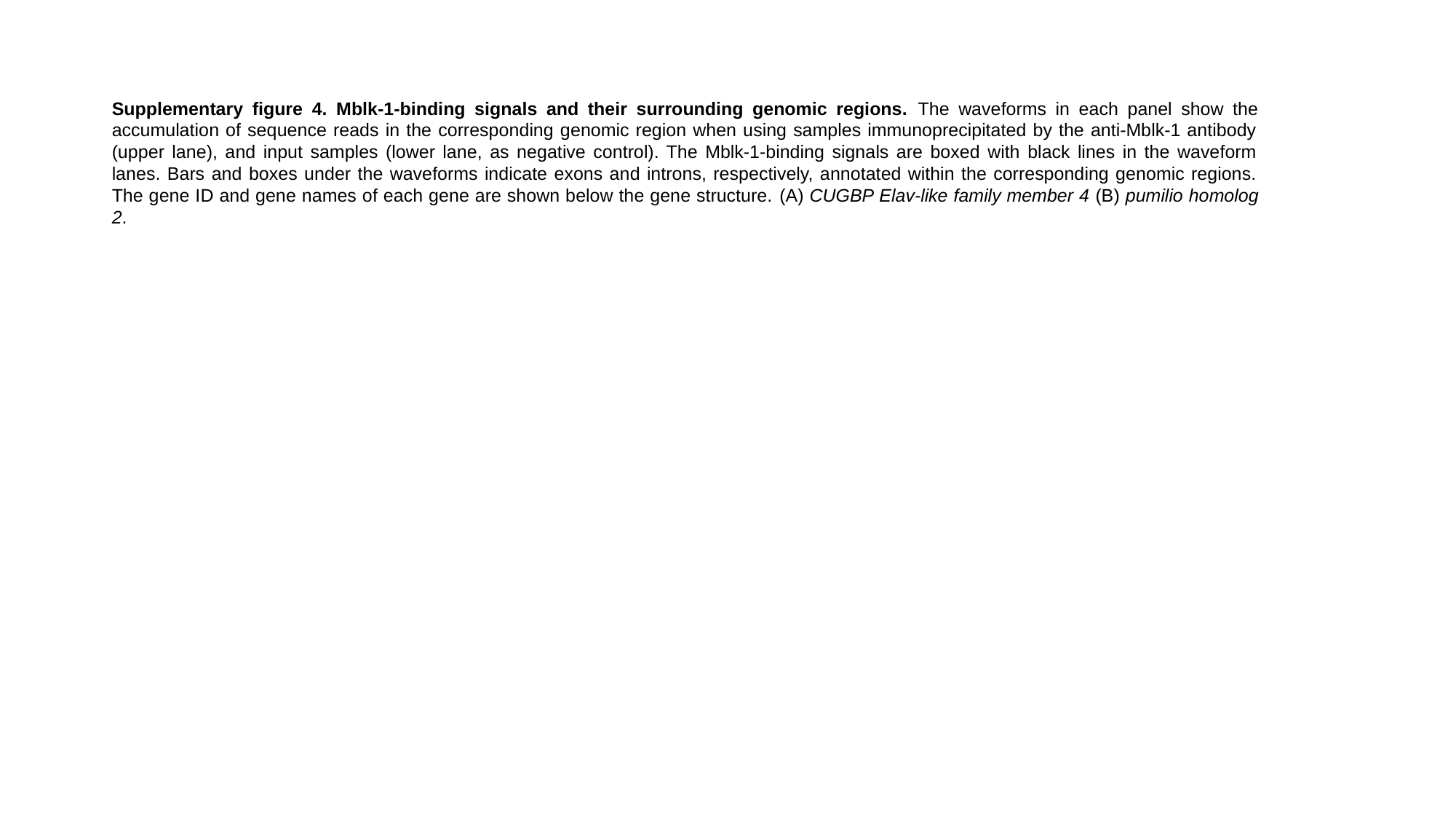

Supplementary figure 4. Mblk-1-binding signals and their surrounding genomic regions. The waveforms in each panel show the accumulation of sequence reads in the corresponding genomic region when using samples immunoprecipitated by the anti-Mblk-1 antibody (upper lane), and input samples (lower lane, as negative control). The Mblk-1-binding signals are boxed with black lines in the waveform lanes. Bars and boxes under the waveforms indicate exons and introns, respectively, annotated within the corresponding genomic regions. The gene ID and gene names of each gene are shown below the gene structure. (A) CUGBP Elav-like family member 4 (B) pumilio homolog 2.

## Slide 9
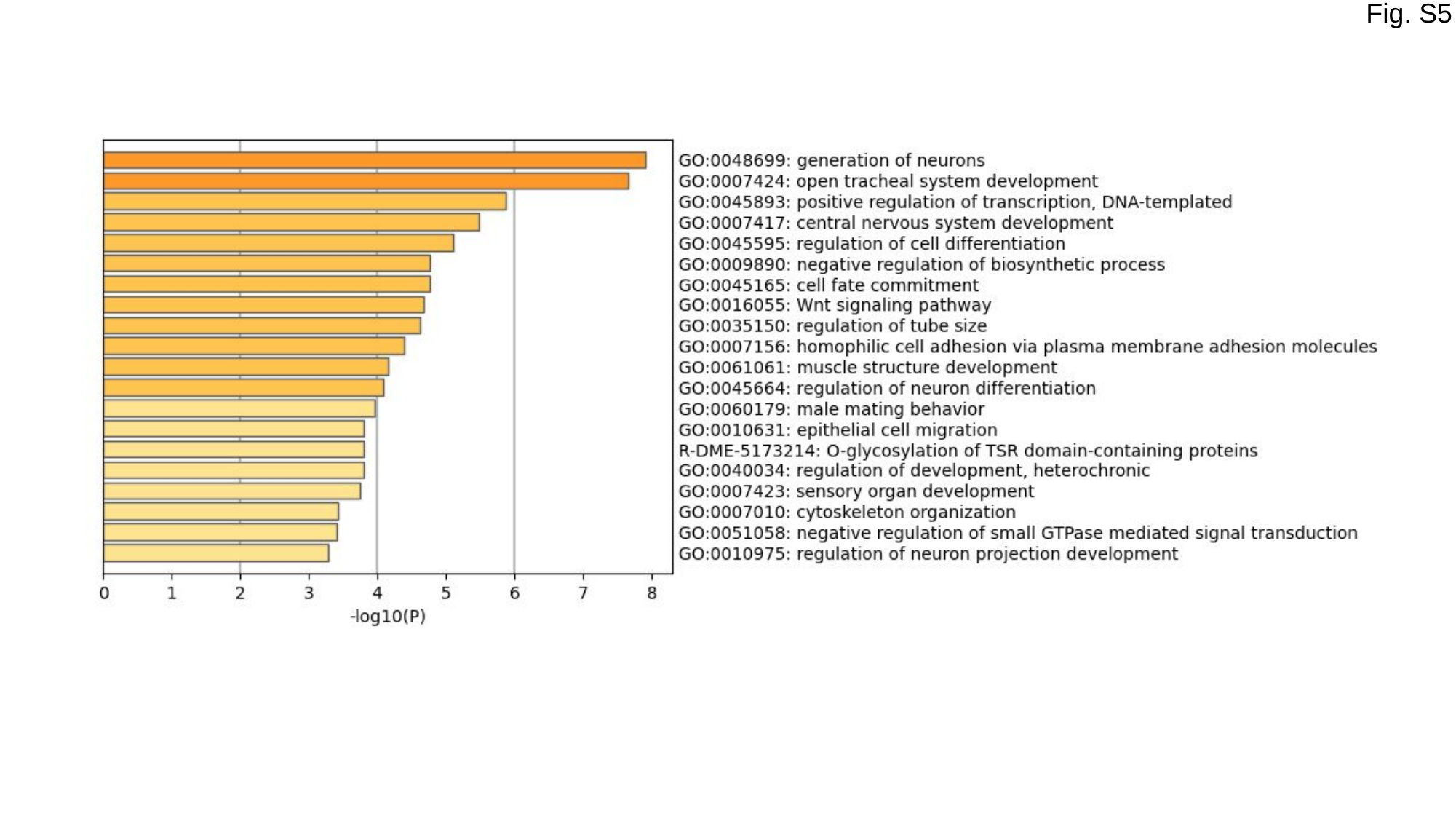

Fig. S5

## Slide 10
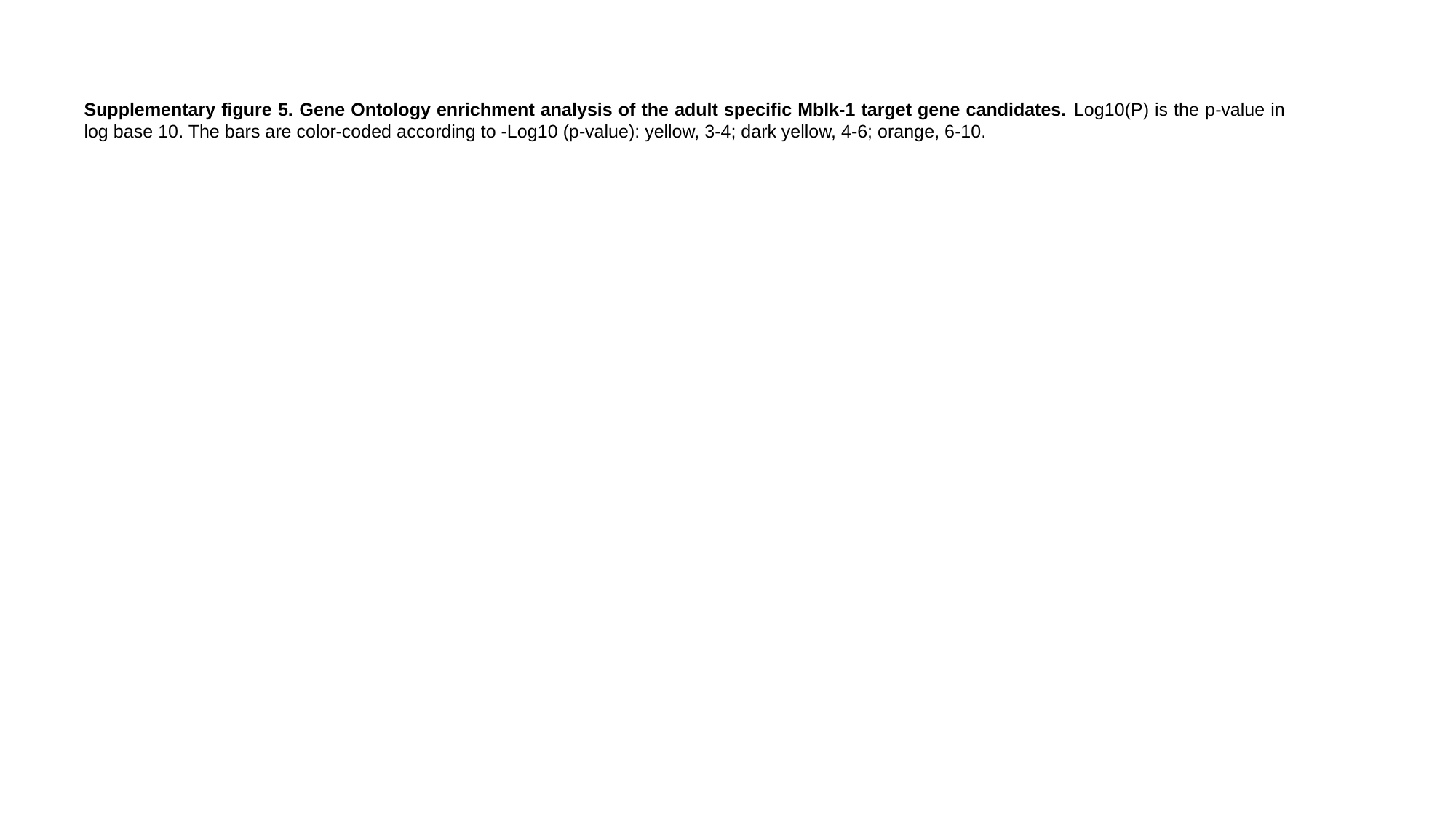

Supplementary figure 5. Gene Ontology enrichment analysis of the adult specific Mblk-1 target gene candidates. Log10(P) is the p-value in log base 10. The bars are color-coded according to -Log10 (p-value): yellow, 3-4; dark yellow, 4-6; orange, 6-10.

## Slide 11
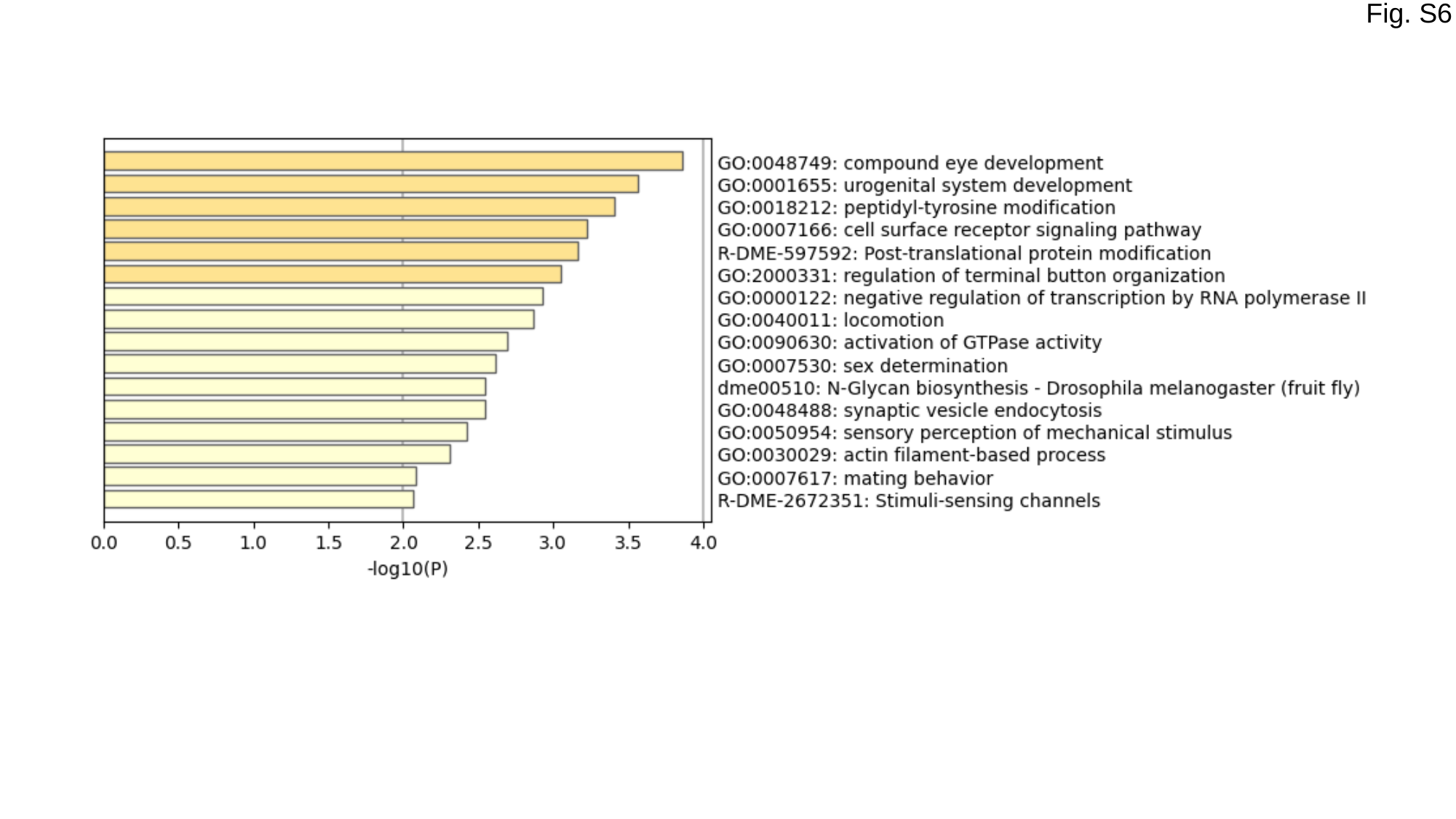

Fig. S6

## Slide 12
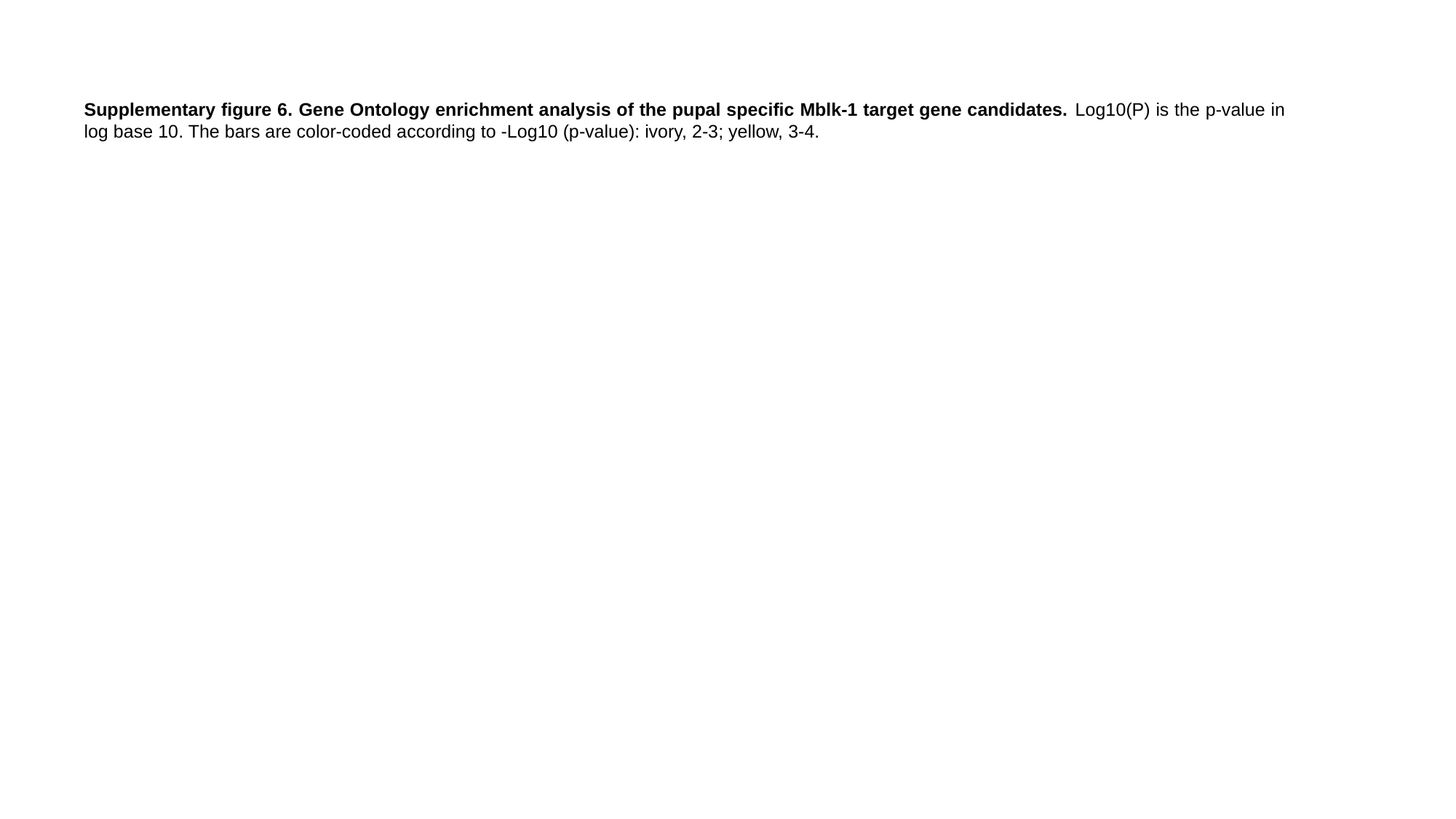

Supplementary figure 6. Gene Ontology enrichment analysis of the pupal specific Mblk-1 target gene candidates. Log10(P) is the p-value in log base 10. The bars are color-coded according to -Log10 (p-value): ivory, 2-3; yellow, 3-4.

## Slide 13
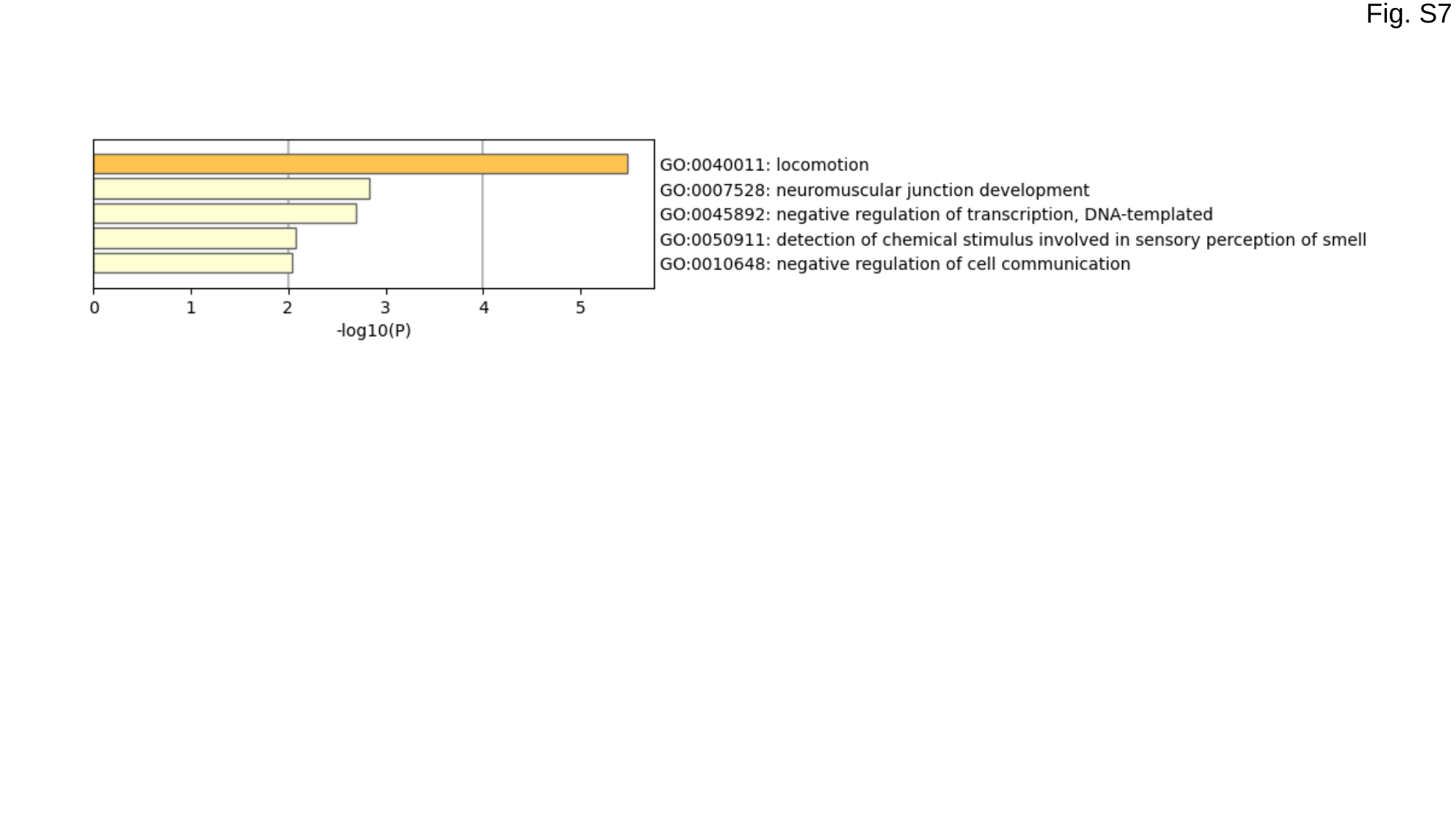

Fig. S7

## Slide 14
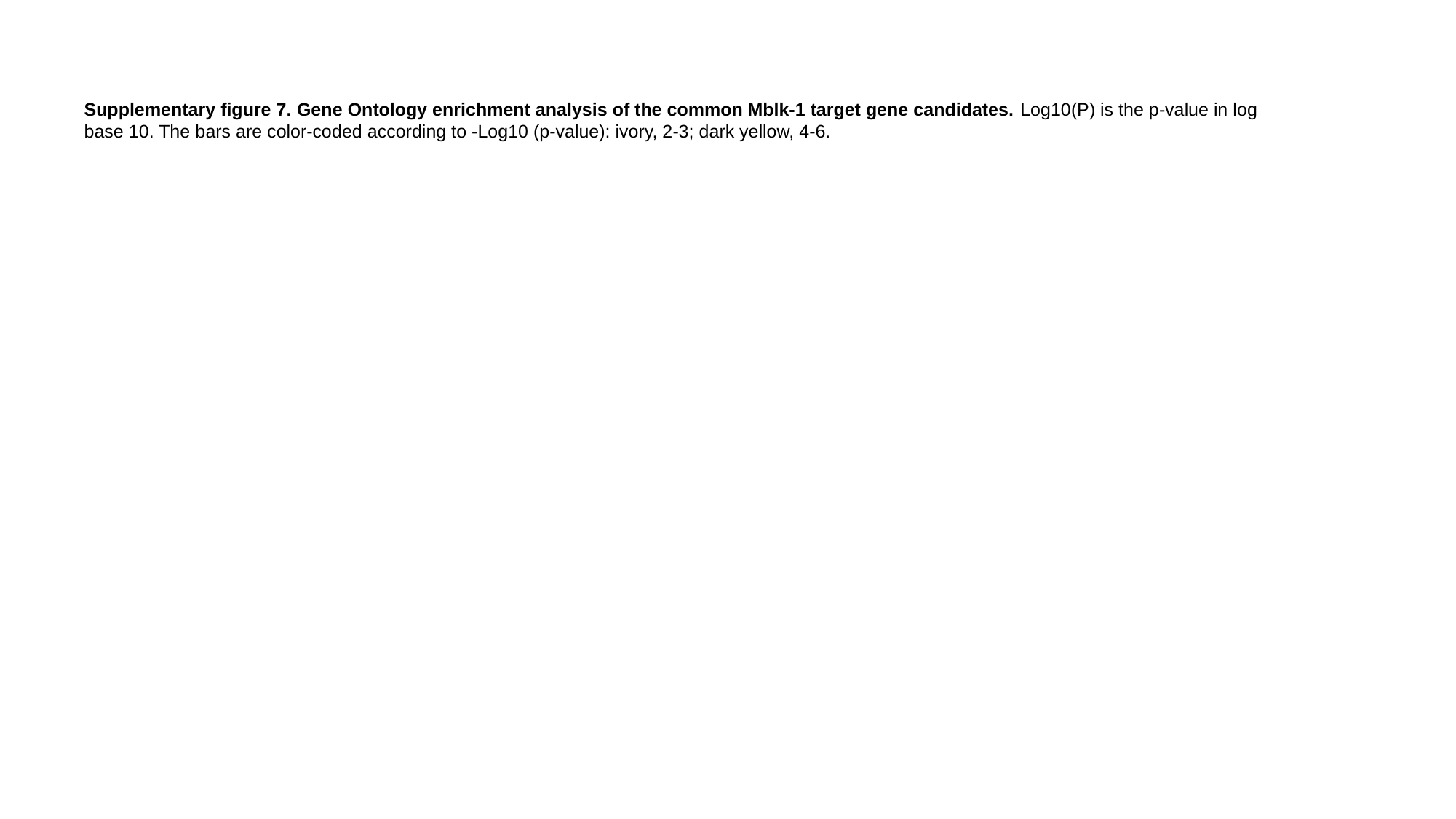

Supplementary figure 7. Gene Ontology enrichment analysis of the common Mblk-1 target gene candidates. Log10(P) is the p-value in log base 10. The bars are color-coded according to -Log10 (p-value): ivory, 2-3; dark yellow, 4-6.

## Slide 15
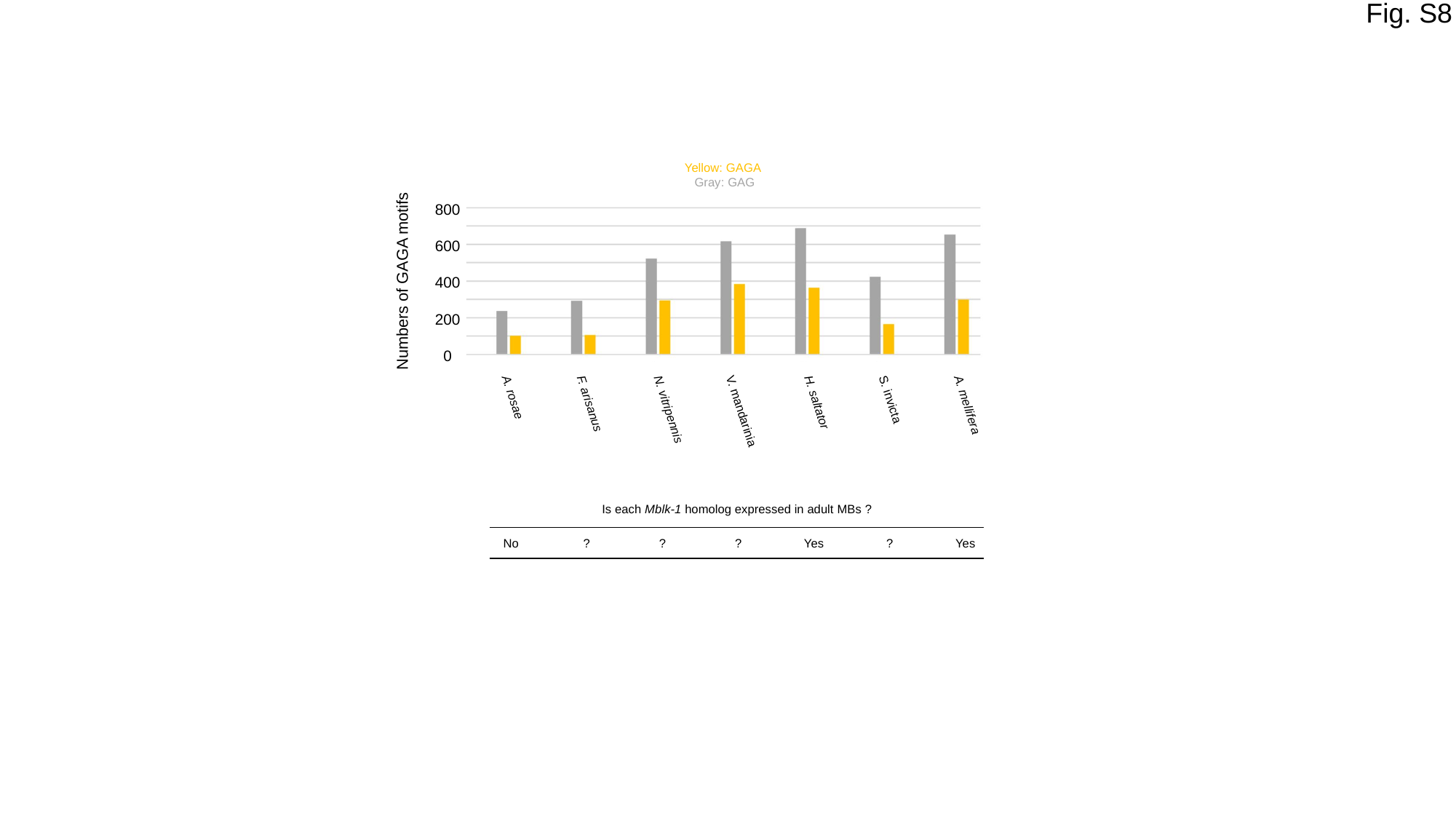

Fig. S8
Yellow: GAGA
Gray: GAG
800
600
Numbers of GAGA motifs
400
200
0
V. mandarinia
S. invicta
F. arisanus
A. rosae
Is each Mblk-1 homolog expressed in adult MBs ?
N. vitripennis
A. mellifera
?
?
?
?
No
Yes
Yes
H. saltator

## Slide 16
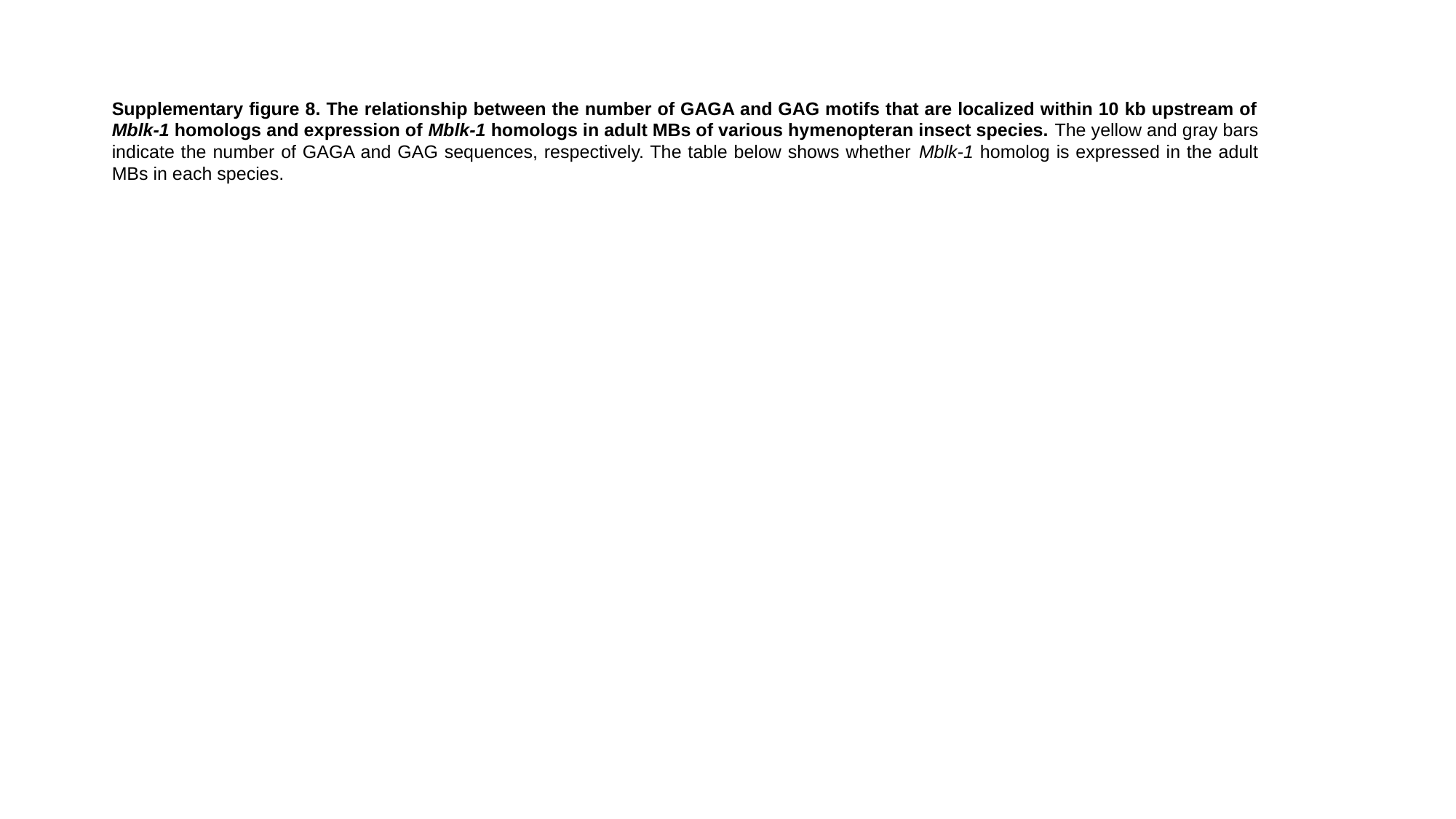

Supplementary figure 8. The relationship between the number of GAGA and GAG motifs that are localized within 10 kb upstream of Mblk-1 homologs and expression of Mblk-1 homologs in adult MBs of various hymenopteran insect species. The yellow and gray bars indicate the number of GAGA and GAG sequences, respectively. The table below shows whether Mblk-1 homolog is expressed in the adult MBs in each species.

## Slide 17
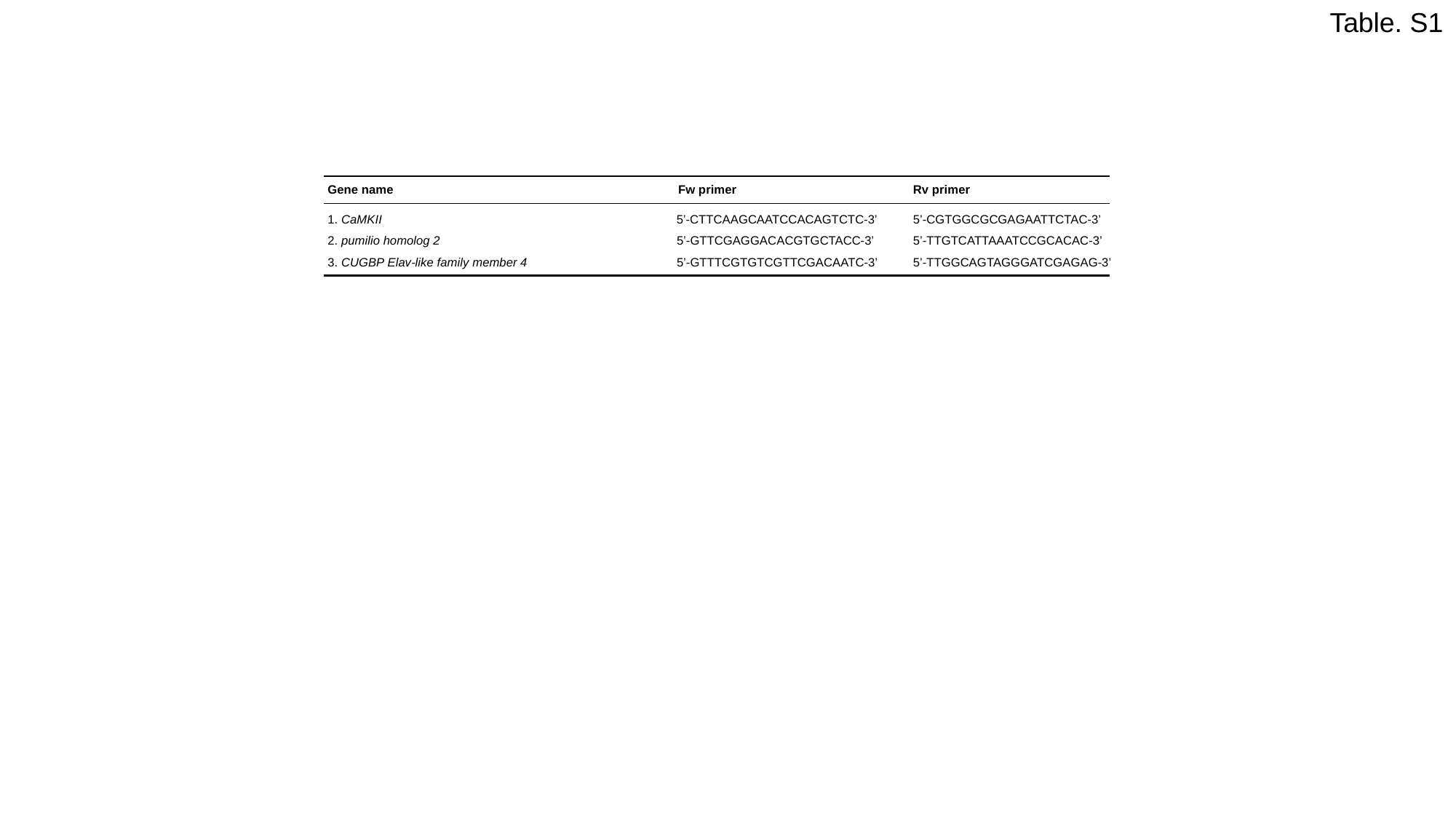

Table. S1
Gene name
Fw primer
Rv primer
1. CaMKII
5’-CTTCAAGCAATCCACAGTCTC-3’
5’-CGTGGCGCGAGAATTCTAC-3’
2. pumilio homolog 2
5’-GTTCGAGGACACGTGCTACC-3’
5’-TTGTCATTAAATCCGCACAC-3’
3. CUGBP Elav-like family member 4
5’-GTTTCGTGTCGTTCGACAATC-3’
5’-TTGGCAGTAGGGATCGAGAG-3’

## Slide 18
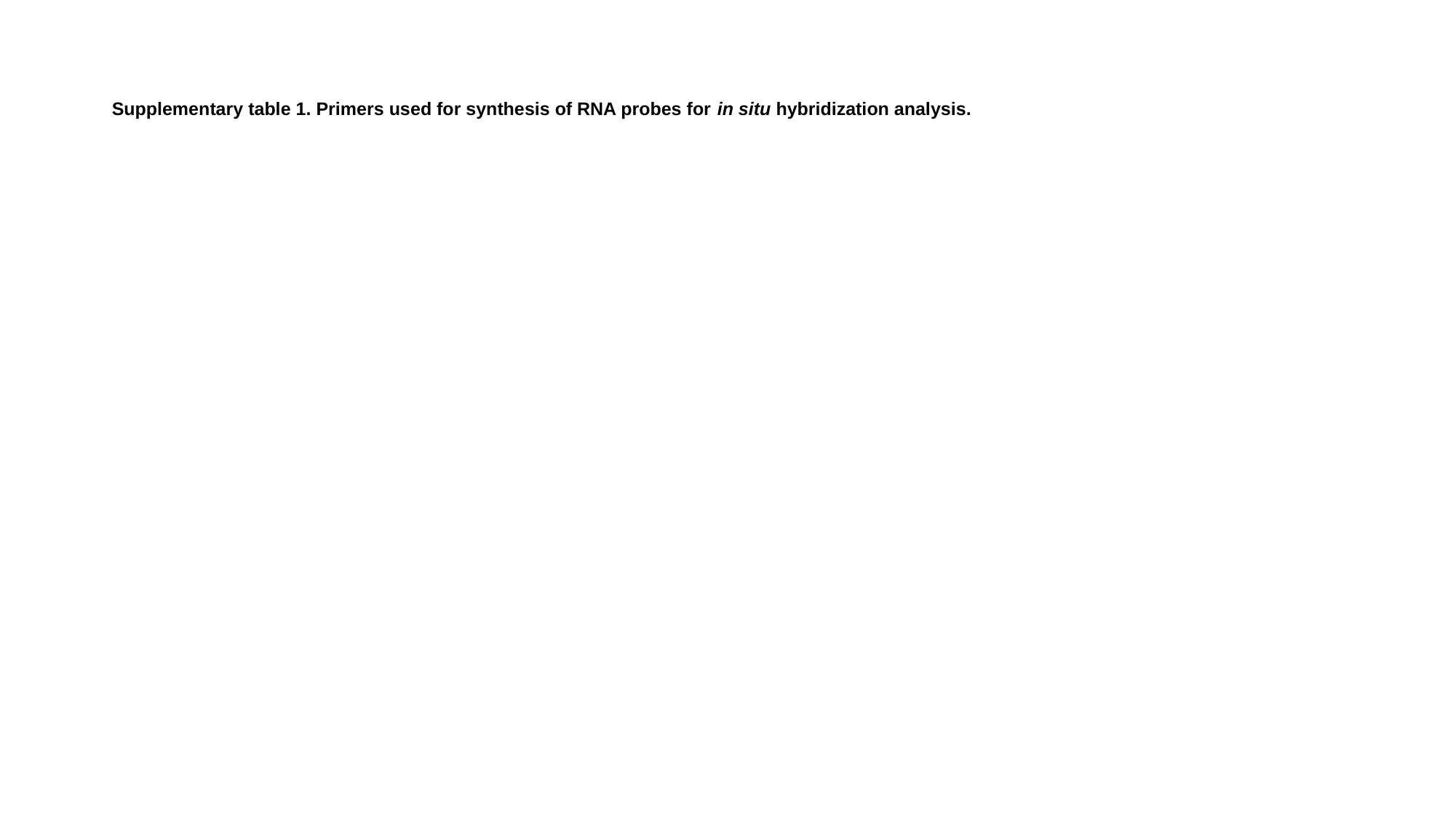

Supplementary table 1. Primers used for synthesis of RNA probes for in situ hybridization analysis.

## Slide 19
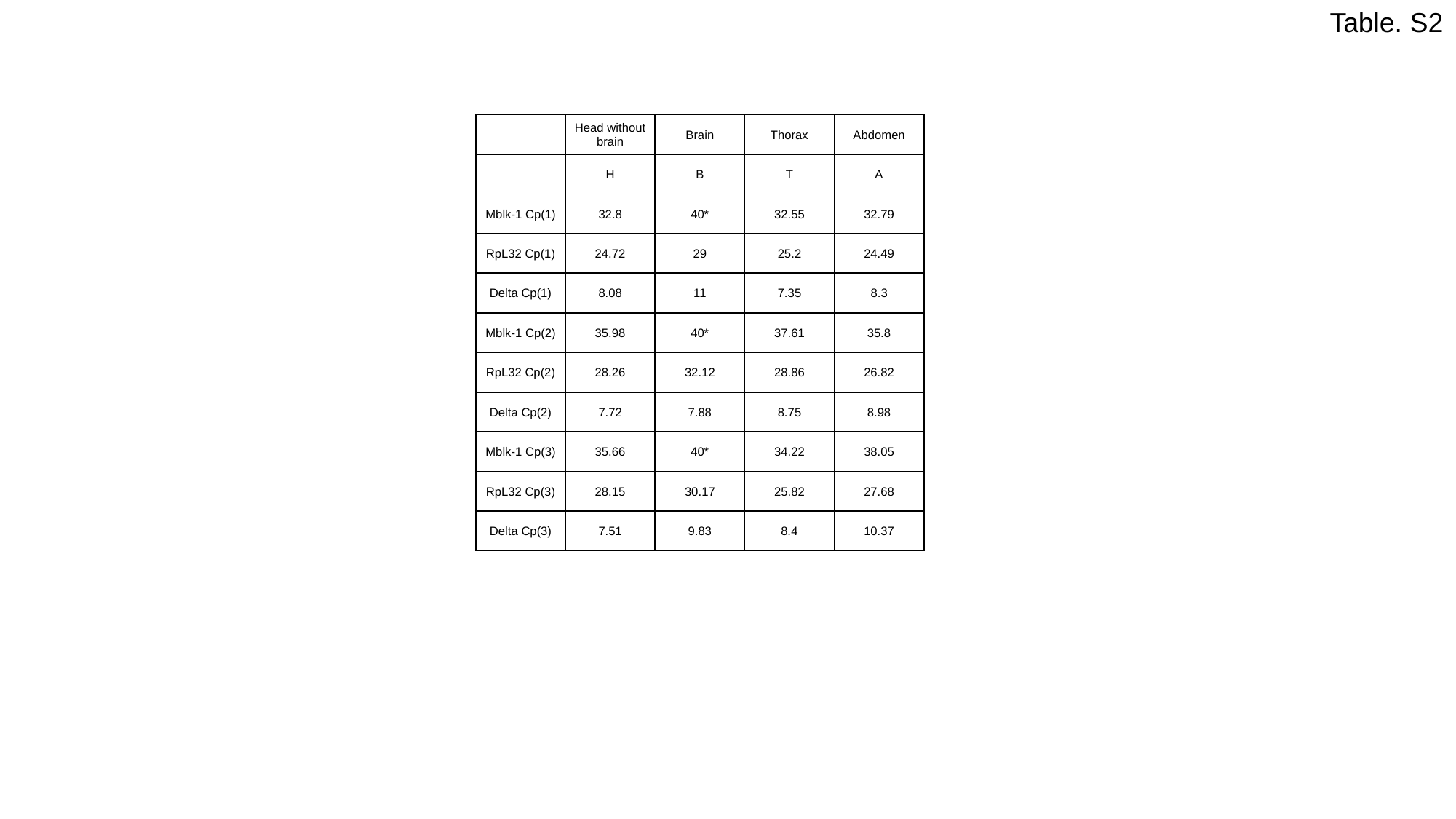

Table. S2
| | Head without brain | Brain | Thorax | Abdomen |
| --- | --- | --- | --- | --- |
| | H | B | T | A |
| Mblk-1 Cp(1) | 32.8 | 40\* | 32.55 | 32.79 |
| RpL32 Cp(1) | 24.72 | 29 | 25.2 | 24.49 |
| Delta Cp(1) | 8.08 | 11 | 7.35 | 8.3 |
| Mblk-1 Cp(2) | 35.98 | 40\* | 37.61 | 35.8 |
| RpL32 Cp(2) | 28.26 | 32.12 | 28.86 | 26.82 |
| Delta Cp(2) | 7.72 | 7.88 | 8.75 | 8.98 |
| Mblk-1 Cp(3) | 35.66 | 40\* | 34.22 | 38.05 |
| RpL32 Cp(3) | 28.15 | 30.17 | 25.82 | 27.68 |
| Delta Cp(3) | 7.51 | 9.83 | 8.4 | 10.37 |

## Slide 20
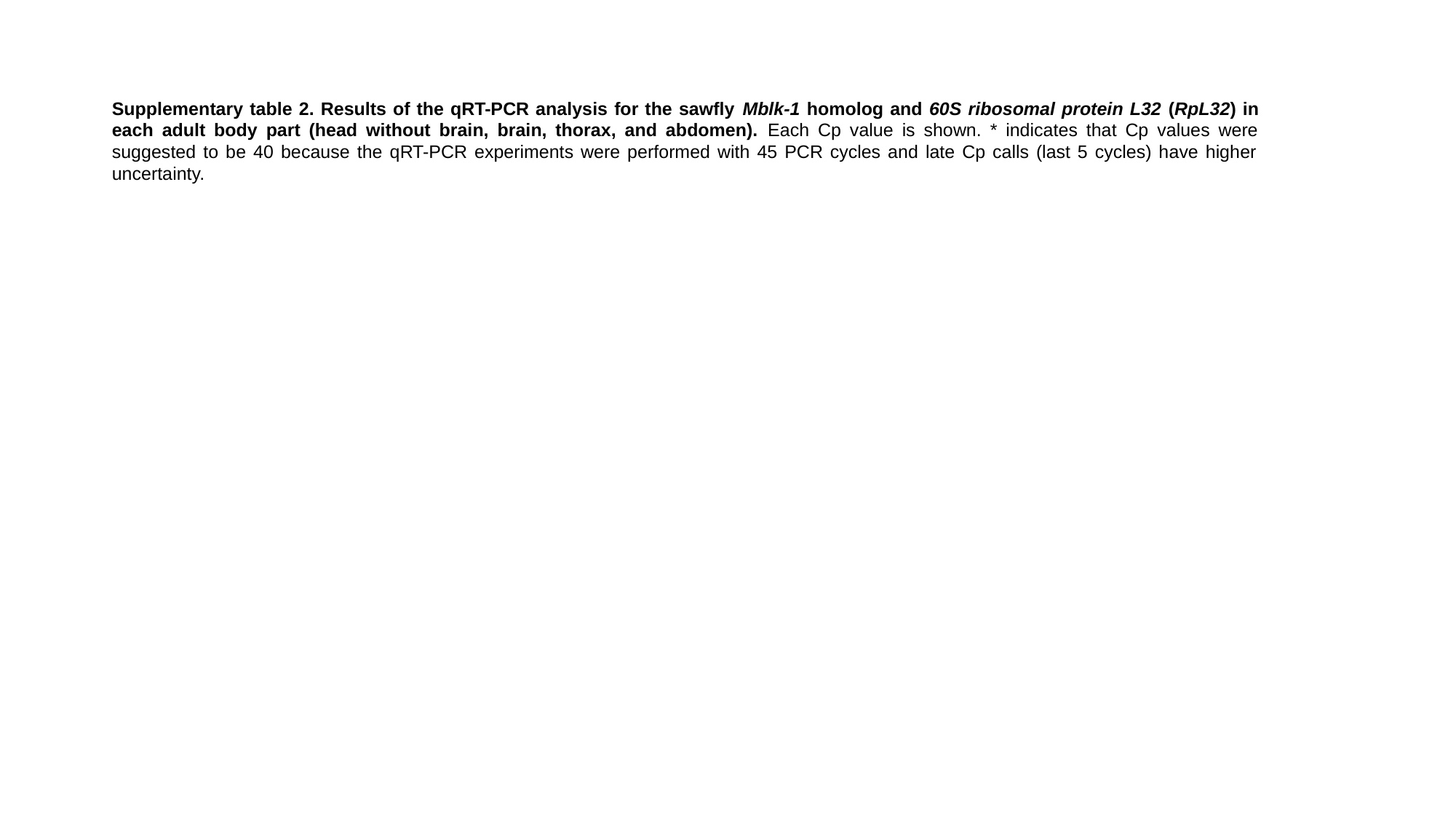

Supplementary table 2. Results of the qRT-PCR analysis for the sawfly Mblk-1 homolog and 60S ribosomal protein L32 (RpL32) in each adult body part (head without brain, brain, thorax, and abdomen). Each Cp value is shown. * indicates that Cp values were suggested to be 40 because the qRT-PCR experiments were performed with 45 PCR cycles and late Cp calls (last 5 cycles) have higher uncertainty.
